# Re-assessment of the taxonomy of Antarctic mushrooms from King George Island, Antarctica

**DOI:** 10.3897/mycokeys.132.191126

**Published:** 2026-05-18

**Authors:** Ji Seon Kim, Jiyun Choi, Min-Soo Choi, Kyung Mo Kim, Jae Eun So, Youngsung Joo, Jaime R. Cabrera-Pardo, Young Woon Lim

**Affiliations:** 1 School of Biological Sciences, Seoul National University, 1 Gwanak-ro, Gwanak-gu, Seoul, Republic of Korea Division of Life Sciences, Korea Polar Research Institute Incheon Republic of Korea; 2 Institute of Biodiversity, Seoul National University, 1 Gwanak-ro, Gwanak-gu, Seoul, Republic of Korea Department of Chemistry, Universidad del Bío-Bío Concepción Chile; 3 Institute for Data Innovation in Science, Seoul National University, 1 Gwanak-ro, Gwanak-gu, Seoul, Republic of Korea Institute of Biodiversity, Seoul National University Seoul Republic of Korea; 4 Division of Life Sciences, Korea Polar Research Institute, 26 Songdomirae-ro, Yeonsu-gu, Incheon, Republic of Korea School of Biological Sciences, Seoul National University Seoul Republic of Korea; 5 Applied and Sustainable Chemistry Laboratory (LabQAS), Department of Chemistry, Universidad del Bío-Bío, Concepción, Chile Institute for Data Innovation in Science, Seoul National University Seoul Republic of Korea; 6 College of Dental Medicine, Roseman University of Health Sciences, South Jordan, USA College of Dental Medicine, Roseman University of Health Sciences South Jordan United States of America

**Keywords:** *
Agaricales
*, amanitin, Divergence time estimation, Multilocus phylogeny, Species delimitation analysis

## Abstract

King George Island, one of the most rapidly warming regions in maritime Antarctica, has experienced pronounced expansion of ice-free areas and proliferation of cryptogamic vegetation in recent decades. This accelerated ecological greening has been accompanied by repeated reports of *Arrhenia*, *Galerina*, and *Omphalina* species. However, despite their frequent occurrence, comprehensive taxonomic assessments of these Antarctic mushrooms remain limited and unresolved. We applied an integrative systematic framework combining morphological examination, multilocus phylogenetic reconstruction, species delimitation analyses, and chemical analyses to 50 mushroom specimens collected from vegetated ice-free areas of King George Island. By integrating polyphasic evidence, we confirmed four species (*Arrhenia antarctica*, *Galerina fallax*, *Galerina venenata*, and *Omphalina frigida*) and resolved several historical taxonomic uncertainties related to these species. This study provides the first phylogenetic analysis of *Arrhenia antarctica*, a first mushroom species documented from Antarctica. Additionally, regarding Antarctic *Omphalina*, for which several new species have recently been reported from Antarctica, we verified that the real species diversity has been inflated and have rectified it. Lastly, LC-MS/MS analysis confirmed amanitin production in materials identified as *Galerina pseudomycenopsis*, thereby clarifying previous taxonomic ambiguity within the *Galerina marginata* species complex by synonymizing *Galerina pseudomycenopsis* with *Galerina venenata*. Divergence-time estimation further suggests that Antarctic lineages diversified before current climate warming, likely in association with repeated glacial-interglacial cycles that reshaped ice-free habitats in Antarctica. By clarifying species identities and integrating multilocus phylogenetics with temporal inference, this study revises the taxonomy of key Antarctic mushrooms and provides a robust framework for future investigation in their diversity, ecological roles, and potential adaptive mechanisms associated with Antarctic greening in extreme environments.

## Introduction

Antarctica is widely regarded as one of the most climate-sensitive regions on Earth and serves as an iconic indicator of anthropogenic climate change. Recent warming has accelerated ice retreat and expanded ice-free areas, leading to measurable increases in terrestrial vegetation cover (Green et al. 2007; Roland et al. 2024). This phenomenon, commonly referred to as Antarctic “greening”, is primarily driven by the proliferation of cryptogamic vegetation such as bryophytes and lichens, along with the two vascular plants *Colobanthus quitensis* and *Deschampsia antarctica* (Longton 1979; Green et al. 2007; Guglielmin et al. 2014; Colesie et al. 2023). These species establish new green habitats that may facilitate the development of novel biotic communities (Green et al. 2007; Duran et al. 2021; Klarenberg et al. 2026). Alongside these environmental changes, reports of agaricoid basidiomata-forming macrofungi (hereafter referred to as mushrooms) have increased significantly. Since the first report in 1956, approximately 20 mushroom species have now been documented from continental Antarctica and adjacent islands (Singer 1956; Horak 1966; Pegler et al. 1980; Gumińska et al. 1994; Putzke and Pereira 1996; Putzke et al. 2012; Garrido-Benavent et al. 2023; Bertazzo-Silva et al. 2024, 2025a, 2025b, 2025c; Bertazzo-Silva and Putzke 2025).

However, taxonomic resolution remains poor for many Antarctic mushrooms due to a lack of comprehensive taxonomic analyses. This problem is particularly pronounced in Antarctic *Arrhenia*, *Galerina*, and *Omphalina*, which are the most frequently reported genera on the continent. Many of these Antarctic mushrooms share noticeable morphological traits, most notably strongly dark-pigmented basidiomata, a character commonly associated with enhanced tolerance to high UV radiation and low temperatures (Krah et al. 2019). *Omphalina antarctica* was originally described in 1956 based solely on morphological characters (Singer 1956). Subsequent phylogenetic studies demonstrated that many *Omphalina* species are appropriately classified within *Arrhenia* (Redhead et al. 2002; Vila 2006; Vila and Llimona 2009; Voitk et al. 2024). As a result, *O. antarctica* was transferred to *Arrhenia* based on the characters of gray-brownish to dark gray omphalinoid basidiomata and non-lichenized but bryophilous traits (Redhead et al. 2002). Despite this revision, only two sequences assigned to *A. antarctica*—one ITS sequence (Accession number PP151300) and one LSU sequence (GQ483368)—are available and neither has undergone a comprehensive taxonomic or phylogenetic evaluation. Given this situation, it is still being published as *A. antarctica* or *O. antarctica* based on morphological characters (Horak 1966; Gumińska et al. 1994; Gyusheva and Chipev 1999; Putzke et al. 2012; Palfner et al. 2020), complicating accurate species identification and subsequent ecological interpretation. Even with recent molecular approaches, taxonomic challenges remain within *Omphalina*. Four Antarctic *Omphalina* species—*O. deschampsiana*, *O. frigida*, *O. ichayoi*, and *O. schaeferi*—have been proposed based on DNA sequence data (Bertazzo-Silva et al. 2025a), but species boundaries remain ambiguous due to weak interspecific morphological differentiation and phylogenetic instability.

Taxonomic challenges are particularly pronounced within the genus *Galerina*. While *G. badipes* and *G. fallax* from Antarctica have been confirmed through phylogenetic evidence (Garrido-Benavent et al. 2023), the identity of Antarctic material previously identified as “*G. marginata*” or *G. pseudomycenopsis* remains unresolved (Putzke and Pereira 1996; Putzke et al. 2012; Garrido-Benavent et al. 2023; Bertazzo-Silva et al. 2024). These two species are part of the problematic *G. marginata* species complex; species delimitation within this species complex has been debated extensively, relying on combinations of morphological, ecological, and amanitin biosynthesis evidence (Groves 1965; Gulden et al. 2005; Landry et al. 2021). However, these criteria often do not correspond with phylogenetic relationships. *Galerina marginata* was originally described as edible, whereas *G. venenata*, the amanitin-producing taxon, has traditionally been treated as a distinct species, despite considerable similarity between the two species (Smith 1953; Groves 1965; Gulden et al. 2005; Landry et al. 2021). *Galerina pseudomycenopsis* has primarily been distinguished from *G. marginata* by its polar distribution rather than molecular evidence (Groves 1965; Gulden 1987; Gulden et al. 2005; Gulden 2010). Phylogenetically, it appears more closely related to the amanitin-producing taxon *G. venenata* than to typical *G. marginata*, yet reports confirming amanitin biosynthesis in *G. pseudomycenopsis* are lacking. Consequently, no significant differences have been observed to clearly distinguish these taxa. Antarctic specimens assigned to “*G. marginata*” exhibit high morphological and ecological similarity to *G. pseudomycenopsis*, yet their precise taxonomic identities remain unresolved without an integrated assessment of morphology, phylogenetic relationships, and amanitin biosynthesis analysis. This ongoing uncertainty underscores the necessity for a polyphasic reassessment to clarify species boundaries within Antarctic *Galerina*.

Beyond taxonomic ambiguities, the evolutionary origins of Antarctic mushrooms continued to be debated. The main issue concerns whether contemporary species represent recent colonists associated with ongoing greening or are remnants of older lineages that survived past climatic transitions (Palfner et al. 2020). Antarctica, formerly part of Gondwana, supported diverse terrestrial vegetation prior to progressive cooling and geographic isolation (McLoughlin 2001; Francis et al. 2008). The development of a continent-wide ice sheet approximately 32–33 million years ago (Mya) resulted in substantial loss of terrestrial biota, leaving an environment characterized by extreme cold, intense ultraviolet radiation, and severe nutrient limitation (Hill and Scriven 1995; McLoughlin 2001; Francis et al. 2008; Barreda et al. 2019). Under these conditions, the long-term survival of most terrestrial macrobiota has been considered unlikely. Nevertheless, recent phylogenetic analyses suggest that Antarctic “*G. marginata*” may have diverged during the Pleistocene, potentially in conjunction with the expansion of cryptogamic vegetation and vascular plants during interglacial periods (Garrido-Benavent et al. 2023). These results prompt further investigation into the timing and ecological context of colonization by other Antarctic mushrooms.

In this study, we focused on mushrooms associated with green habitats in ice-free areas along Maxwell Bay, King George Island (KGI), a region particularly sensitive to climate change. Our study sites included the Fildes Peninsula (the largest ice-free area on KGI) and two ice-free enclaves along the island’s margins, where South Korea’s King Sejong Station is located nearby: Barton Peninsula and Weaver Peninsula. Based on previous studies, three species (*Arrhenia antarctica*, *Galerina fallax*, and *Omphalina pyxidata*) have been reported from these areas (Pegler et al. 1980; Putzke and Pereira 1996; Palfner et al. 2020). While vegetation studies in these areas have advanced considerably (Kim et al. 2007; Lee et al. 2009; Parnikoza et al. 2015; So et al. 2023; Roland et al. 2024), taxonomic and ecological assessments of associated mushrooms have remained fragmentary and unsystematic. To address these gaps, we conducted a comprehensive reassessment of Antarctic mushroom diversity using a polyphasic approach integrating morphological analysis, phylogenetic analyses, species delimitation analyses, and chemical profiling (amanitin) for selected species. As Antarctic mushrooms have adapted and reproduced under Antarctic extremes, they would offer direct insight into fungal survival in extreme habitats and hold potential as sensitive microbial indicators of climate-driven ecological change in Antarctica.

## Methods

### Field sampling and sample treatment

Fungal specimens were collected from the Barton, Fildes, and Weaver Peninsulas on King George Island, Antarctica, between 2022 and 2025. Fresh basidiomata were photographed in the field to document macromorphological characters prior to preservation. As sampling was conducted over multiple years under varying experimental conditions, pretreatment procedures were adjusted according to the requirements of subsequent analyses. For DNA extraction, whole or sectioned basidiomata were preserved in absolute ethanol or 20% glycerol in the field and stored at -20 °C until laboratory processing. For morphological observation, basidiomata were dried either at room temperature by pressing or by freeze-drying. Dried basidiomata were then placed in individual paper envelopes with silica gel desiccants. In addition to Antarctic specimens, a Korean specimen of *Galerina marginata* (SFC20140530-09) from the fungal collection at Seoul National University was included as a reference taxon for species delimitation and divergence-time estimation analyses. Detailed collection information for all specimens is provided in Suppl. material 1.

### Molecular analyses (DNA extraction, PCR, and sequencing)

Tissue samples of approximately 0.5 cm^2^, obtained from either dried specimens or ethanol-fixed tissues, were used for genomic DNA extraction with the AccuPrep Genomic DNA extraction kit (Bioneer Co., Daejeon, Republic of Korea). Before proceeding with the manufacturer’s protocol, tissues were immersed in TL buffer and homogenized using a Bead Ruptor Elite homogenizer (OMNI International, Kennesaw, GA, USA). Subsequent steps were performed according to the manufacturer’s instructions.

PCR amplification was performed using AccuPower PCR premix (Bioneer Co., Daejeon, Republic of Korea) on a C1000 thermal cycler (Bio-Rad, Richmond, CA, USA). The internal transcribed spacer (ITS) region was amplified with the primer pair ITS1F and ITS4B (Gardes and Bruns 1993) under the following conditions: 95 °C for 5 min; 35 cycles of 95 °C for 40 s, 55 °C for 40 s, and 72 °C for 1 min; followed by 72 °C for 5 min. The large subunit (LSU) region was amplified using the LR0R and LR5 primer pair (Vilgalys and Hester 1990) under the same conditions as ITS region amplification. RNA polymerase II (*RPB2*) region was amplified using the primers RPB2-6F and RPB2-7.1R (Matheny 2005) under the same conditions described in Landry (2019). If *RPB2* amplification was insufficient, nested PCR was performed using bRPB2-7R (Matheny 2005), as described by Landry (2019). Amplification was verified by electrophoresis on a 1% agarose gel, and PCR products were purified using ExoSAP-IT^TM^ PCR Product Cleanup Reagent (Thermo Fisher Scientific, Waltham, MA, USA). Sequencing was conducted with the same primer sets as used for PCR on an ABI Prism 3700 Genetic Analyzer (Life Technologies, Gaithersburg, MD, USA) at Macrogen (Seoul, Republic of Korea).

### Morphological analysis

Macromorphological characters were described from field photographs of fresh basidiomata, and color descriptions (names and alphanumeric codes) were assigned according to the Methuen Handbook of Colour (Kornerup and Wanscher 1967). Microscopic features, including basidiospores, basidia, and additional structures, were observed using dried specimens mounted in 5% KOH and stained with 1% phloxine B. Observations were conducted with an Eclipse 80i compound light microscope (Nikon, Japan) equipped with NIS-Elements BR software v3.2 (Nikon, Japan). A minimum of 20 basidia, 30 basidiospores, and 20 individuals of each additional structure were examined and measured at 400× magnification using Image J (Abràmoff et al. 2004). Basidiospore measurements included the E value, defined as the ratio of spore length to width, and the Q value, representing the mean E value.

### Phylogenetic analysis

The generated sequences were trimmed using the *Trim Ends* function in Geneious Prime software v2026.0.2 (https://www.geneious.com) and manually edited as required. Forward and reverse reads were assembled using the *de novo* assembly function in Geneious Prime software v2026.0.2, and all newly generated sequences were deposited in GenBank (Table 1). Genus-level identifications were initially performed via NCBI BLAST searches using the ITS sequences, followed by the selection and inclusion of additional genetic markers to enhance species-level resolution. Reference sequences, including those derived from holotypes and other reliable sources, were retrieved from GenBank and UNITE and incorporated into the phylogenetic analyses with the newly generated sequences (Suppl. material 2). Each locus was aligned using MAFFT v7 with the “L-INS-I” option (Katoh et al. 2019). Because many publicly available ITS sequences contained only partial regions (such as ITS2 or 5.8S), ITSx v1.1.3 with the options *-t* “F” and *-save_regions* “all” was used to split ITS1, 5.8S, and ITS2 prior to alignment (Bengtsson‐Palme et al. 2013). The dataset of each section was aligned respectively, and the three resulting alignments were concatenated to form a complete ITS dataset. For *RPB2*, most sequences lacked intron regions, so only exon regions were included in the analysis. The alignments for ITS, LSU, and *RPB2* were concatenated to generate a multilocus dataset for each genus. Phylogenetic trees were inferred using RAxML-HPC with 1,000 bootstrap replicates (Stamatakis 2014). All alignments used for phylogenetic inference were deposited in figshare (https://figshare.com/articles/dataset/_/31952088).

**Table 1.** Information on specimens examined in this study. Locality abbreviations: BP = Barton Peninsula, FP = Fildes Peninsula, KGI = King George Island, NI = Nelson Island, WP = Weaver Peninsula.

Species	Specimen	Country	Locality	ITS	LSU	RPB2
*Arrhenia antarctica*	KOPRI-MU00004	Antarctica	BP	PZ052155	PZ052202	―
	KOPRI-MU00014	Antarctica	BP	PZ052156	PZ052203	PZ098639
	KOPRI-MU00018	Antarctica	NI	PZ052157	PZ052204	PZ098640
	SFC20220210-02	Antarctica	BP	PZ052158	PZ052205	PZ098641
	SFC20220213-01	Antarctica	BP	PZ052159	PZ052206	PZ098642
	SFC20230121-01	Antarctica	BP	PZ052160	PZ052207	PZ098643
	SFC20230125-01	Antarctica	BP	PZ052161	PZ052208	―
	SFC20230125-02	Antarctica	BP	PZ052162	―	PZ098644
	SFC20230125-06	Antarctica	BP	PZ052163	PZ052209	PZ098645
	SFC20230125-07	Antarctica	BP	PZ052164	PZ052210	―
	SFC20230306-01	Antarctica	FP	PZ052165	―	PZ098646
	SFC20230309-01	Antarctica	FP	PZ052166	―	PZ098647
	SFC20230309-02	Antarctica	FP	PZ052167	―	―
	SFC20230314-01	Antarctica	FP	PZ052168	―	―
	SFC20230314-02	Antarctica	FP	PZ052169	―	PZ098648
	SFC20250121-01	Antarctica	BP	PZ052170	PZ052211	PZ098649
	SFC20250125-01	Antarctica	BP	PZ052171	PZ052212	―
	SFC20250125-03	Antarctica	BP	PZ052172	PZ052213	PZ098650
	SFC20250126-01	Antarctica	WP	PZ052173	PZ052214	PZ098651
	SFC20250207-01	Antarctica	BP	PZ052174	PZ052215	PZ098652
	SFC20250207-02	Antarctica	BP	PZ052175	PZ052216	PZ098653
*Galerina fallax*	KOPRI-MU00007	Antarctica	BP	PZ052176	PZ052217	PZ098680
	KOPRI-MU00015	Antarctica	BP	PZ052177	PZ052218	PZ098677
	SFC20220220-01	Antarctica	BP	PZ052178	PZ052219	PZ098678
	SFC20230123-02	Antarctica	BP	PZ052179	PZ052220	―
	SFC20230125-05	Antarctica	BP	PZ052180	PZ052221	PZ098679
*Galerina marginata*	SFC20140530-09	Republic of Korea	Jeonbuk State	KX773866	―	PZ098676
*Galerina venenata*	KOPRI-MU00003	Antarctica	BP	PZ052181	PZ052222	PZ098673
	KOPRI-MU00006	Antarctica	BP	PZ052182	PZ052223	PZ098674
	KOPRI-MU00013	Antarctica	BP	PZ052183	PZ052224	PZ098675
*Omphalina frigida*	KOPRI-MU00002	Antarctica	BP	PZ052184	PZ052225	PZ098654
	KOPRI-MU00005	Antarctica	BP	PZ052185	PZ052226	PZ098658
	KOPRI-MU00009	Antarctica	BP	PZ052186	PZ052227	PZ098655
	KOPRI-MU00010	Antarctica	BP	PZ052187	PZ052228	―
	KOPRI-MU00011	Antarctica	BP	PZ052188	PZ052229	PZ098656
	KOPRI-MU00012	Antarctica	BP	PZ052189	PZ052230	PZ098659
	KOPRI-MU00016	Antarctica	BP	―	PZ052231	PZ098661
	KOPRI-MU00017	Antarctica	NI	―	PZ052232	PZ098660
	KOPRI-MU00019	Antarctica	NI	PZ052190	PZ052233	PZ098657
	SFC20220210-01	Antarctica	BP	PZ052191	PZ052234	PZ098668
	SFC20220219-01	Antarctica	BP	PZ052192	PZ052235	PZ098664
	SFC20230121-02	Antarctica	BP	―	PZ052236	PZ098662
	SFC20230123-01	Antarctica	BP	PZ052193	―	―
	SFC20230307-01	Antarctica	KGI	PZ052194	―	PZ098671
	SFC20230314-03	Antarctica	KGI	PZ052195	―	PZ098672
	SFC20230319-01	Antarctica	KGI	PZ052196	―	PZ098669
	SFC20250125-02	Antarctica	BP	PZ052197	PZ052237	PZ098665
	SFC20250125-04	Antarctica	BP	PZ052198	PZ052238	PZ098667
	SFC20250125-05	Antarctica	BP	PZ052199	PZ052239	PZ098663
	SFC20250207-03	Antarctica	BP	PZ052200	PZ052240	PZ098666
	SFC20250207-04	Antarctica	BP	PZ052201	PZ052241	PZ098670

### Species delimitation analysis

Species boundaries were evaluated using three delimitation approaches, each applied to a specific genus. Three single-locus methods, Automatic Barcode Gap Discovery (ABGD) (Puillandre et al. 2012), General Mixed Yule Coalescent (GMYC) (Fujisawa and Barraclough 2013), and a Poisson Tree Processes (PTP) (Zhang et al. 2013; Kapli et al. 2017) were implemented on the same dataset generated for phylogenetic analysis. Specimens lacking full-length sequences for any of the target loci were excluded from the analyses to minimize biases in genetic distance estimation caused by uneven sequence coverage. For *Arrhenia* and *Omphalina*, LSU and *RPB2* were not included in the analyses because sequence data for these markers were unavailable for most specimens.

ABGD analyses were conducted using the iTaxoTools web interface (https://itaxotools.org/index.html, accessed on 1 October 2025) (Vences et al. 2021) under the Kimura 2-parameter model with the default parameters (Puillandre et al. 2012). PTP analyses were performed on the PTP web server (https://species.h-its.org/ptp/) (Zhang et al. 2013), utilizing RAxML phylogenetic trees as input. GMYC analyses were carried out in R packages *ape* (Paradis and Schliep 2019) and *splits* (Fujisawa and Barraclough 2013) on ultrametric trees generated in BEAST v2.7.7 (Bouckaert et al. 2019), using a site heterogeneity model estimated by the built-in BEAST model test and a log normal relaxed clock model (Drummond et al. 2006) for 100 million Monte Carlo Markov Chains (MCMC), sampled every 5,000 generations. Convergence was evaluated by effective sample size (ESS) values greater than 200 in Tracer v1.7.2 (Rambaut et al. 2018). Prior to species delimitation analyses, the resulting trees were summarized into a maximum clade credibility (MCC) tree for each genus using TreeAnnotator v1.7.2 with a 10% burn-in (Bouckaert et al. 2019).

### Divergence-time estimation

Divergence times were estimated using BEAST v2.7.7, employing priors established in previous studies (Sánchez‐Ramírez et al. 2015; Zhao et al. 2016; Zhao et al. 2017) and a three-gene dataset (ITS+LSU+*RPB2*) comprising 50 taxa from eight families of *Agaricales*, two families of *Amylocorticiales*, two of *Hymenochaetales*, and one of *Russulales*. An uncorrelated lognormal relaxed clock model was implemented (Drummond et al. 2006; Lepage et al. 2007), with independently estimated substitution rates for each partition. Calibration was based on two gamma-distributed priors: *Agaricales* (offset = 90 Ma, scale = 20, shape = 1) and *Hymenochaetales* (offset = 113 Ma, scale = 20, shape = 1) (Sánchez‐Ramírez et al. 2015; Zhao et al. 2016). Analyses were conducted for 700 million MCMC generations, and chain convergence (ESS > 200) was assessed using Tracer v1.7.2 (Rambaut et al. 2018). MCC trees were generated with TreeAnnotator v1.7.2, applying a 10% burn-in (Bouckaert et al. 2019), and 95% highest posterior density (HPD) intervals were visualized on the ultrametric tree. Geological timescales were incorporated into the ultrametric tree using the “geoscalePhylo” function from the *strap* package (Bell and Lloyd 2015) in R v4.3.2. The RAxML phylogenetic tree and the ultrametric tree were compared, and bootstrap values from the RAxML phylogenetic tree were mapped to the corresponding nodes of the ultrametric tree using the *ape* (Paradis and Schliep 2019) and *geiger* (Harmon et al. 2008) packages in R v4.3.2.

### Quantification of amanitins

The standard compound of α-amanitin was purchased for analysis (A2263, Sigma-Aldrich, USA). The following other compounds used for the analysis of amanitin were purchased and used; Water (8585-3904, Daejung Chemicals & Metals Co., Republic of Korea), methyl alcohol LCMS grade (67-56-1, Samchun Chemicals, Republic of Korea), and acetonitrile LCMS grade (75-05-8, Samchun Chemicals, Republic of Korea), ammonium acetate HPLC grade (A639-500, Thermo Fisher Scientific, USA), and acetic acid optima LCMS (A113-50, Thermo Fisher Scientific, USA).

The quantity of amanitins in the ethanol preservative used for the storage of *Galerina* tissues was measured by liquid chromatography-mass spectrometry. The analytic conditions were modified from a previously reported protocol (Bambauer et al. 2020). Specifically, the ethanol preservatives were filtered by 0.22 µm PTFE syringe filter and injected to Ultimate 3000 liquid chromatography (Thermo Fisher Scientific, United States). Separation was performed using a Waters C18 CSH column (100 × 2.1 mm I.D., particle size 1.7 µm). Mobile phase A (water 8 mM ammonium acetate and 0.05% acetic acid) and mobile phase B (ACN and MeOH in equal shares, with 1% water, 4 mM ammonium acetate, and 0.05% acetic acid) were used in following gradient: 0 min, 1% B; 1 min, 1% B; 2 min, 15% B; 6 min, 30% B; 7.5 min, 99% B; 8.5 min, 99% B; 8.6 min, 1% B; 14 min, 1% B. The flow rate was set to 0.4 ml/min, and the column oven temperature was maintained at 40 °C.

The ionization was performed in positive polarity of electrospray ionization with the following parameters: sheath gas flow 60, aux gas flow 10, spray voltage 4 kV, capillary temperature 320 °C. The mass spectra were acquired using t-SIM and PRM modes. The t-SIM mode parameters were set as follows: resolution 70,000, AGC target 5e4, maximum IT 200 ms, isolation window 1.0 m/z. Parameters for PRM mode were set as follows: default charge 1, resolution 17,500, AGC target 2e5, maximum IT 100 ms, isolation window 1.0 m/z, first fixed mass 80 m/z, NCE 28%. By identifying MS1, molecular formula, and fragment ion of m/z 259.1287 of β-amanitin, we also quantified this feature as putative β-amanitin. The inclusion list contained α-amanitin (m/z 919.36144) and β-amanitin (m/z 920.34546), for their monoprotonated ions. Fragment ion of m/z 259.1287 was monitored for quantification of both α- and β-amanitin.

## Results

### Phylogenetic analysis and species delimitation

**Figure 1.**
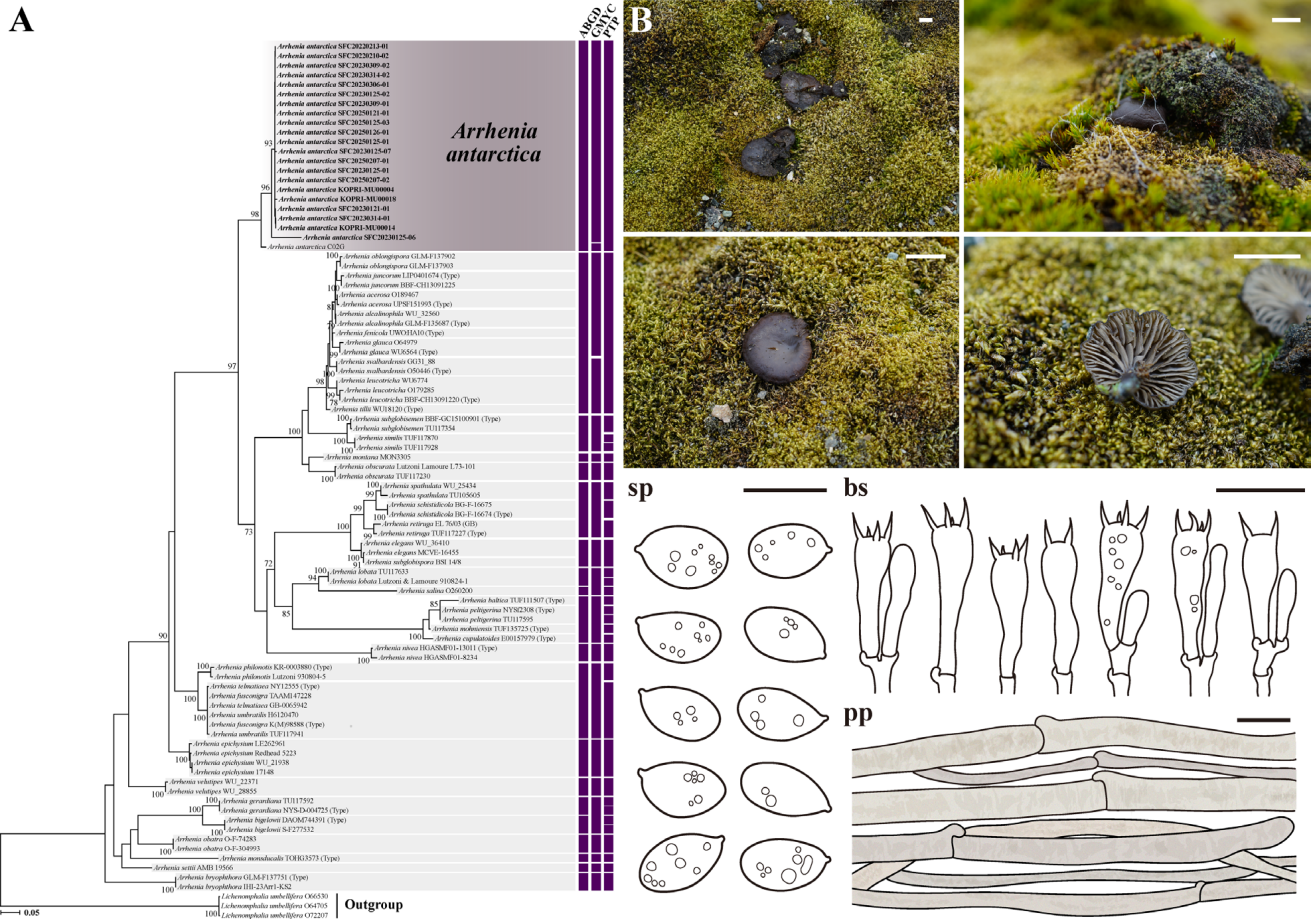
Phylogenetic tree, species delimitation results, and morphological characterization of Antarctic *Arrhenia antarctica*. **A**. Maximum likelihood phylogenetic tree of *Arrhenia* based on ITS and LSU sequences. Colored bars adjacent to the phylogeny represent putative species boundaries determined by ABGD, GMYC, and PTP analyses. Bootstrap support values over 70 are shown at nodes; **B**. Morphological characters of Antarctic *Arrhenia antarctica* with field photographs of basidiomata and line drawings of microscopic structures. Abbreviations are as follows: sp = basidiospores, bs = basidia, pp = pileipellis. Scale bars: 1 cm (basidiomata); 10 μm (sp); 20 μm (bs, pp).

**Figure 2.**
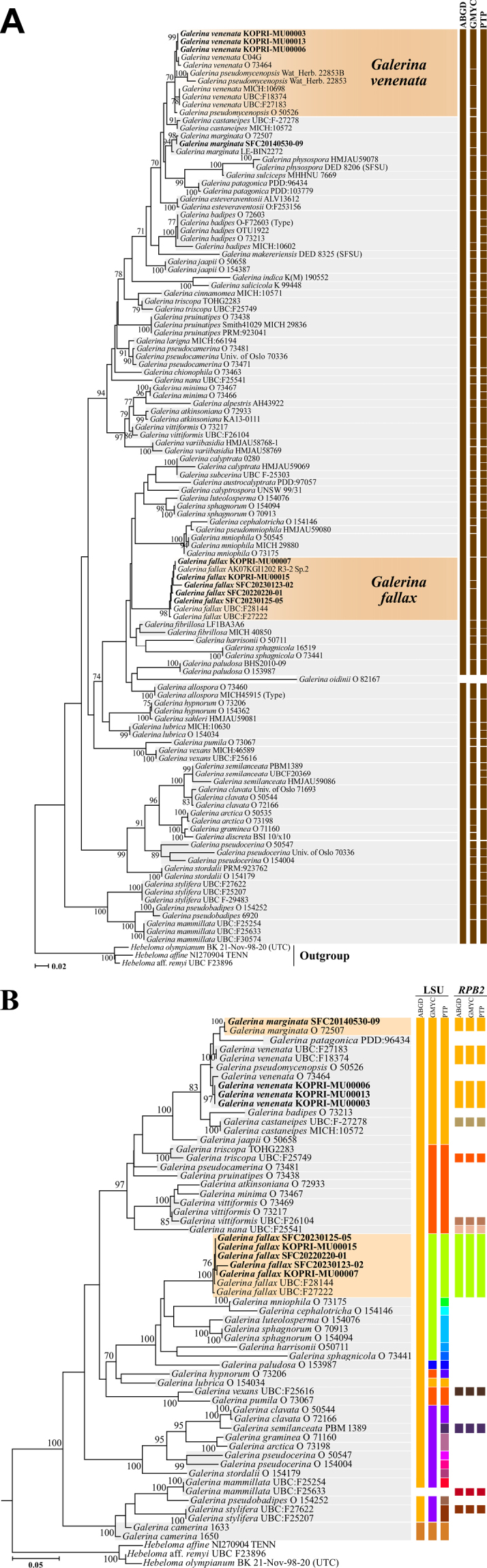
Phylogenetic tree and species delimitation results of Antarctic *Galerina*. Colored bars adjacent to the phylogeny represent putative species boundaries from ABGD, GMYC, and PTP analyses. Bootstrap support values over 70 are shown at nodes. **A**. ITS-based species delimitation results shown onto the ITS+LSU+*RPB2*-based RAxML phylogeny including all *Galerina* specimens; **B**. LSU/*RPB2*-based species delimitation results mapped onto the ITS+LSU+*RPB2*-based RAxML phylogeny including specimens with sequence data available for at least two loci.

**Figure 3.**
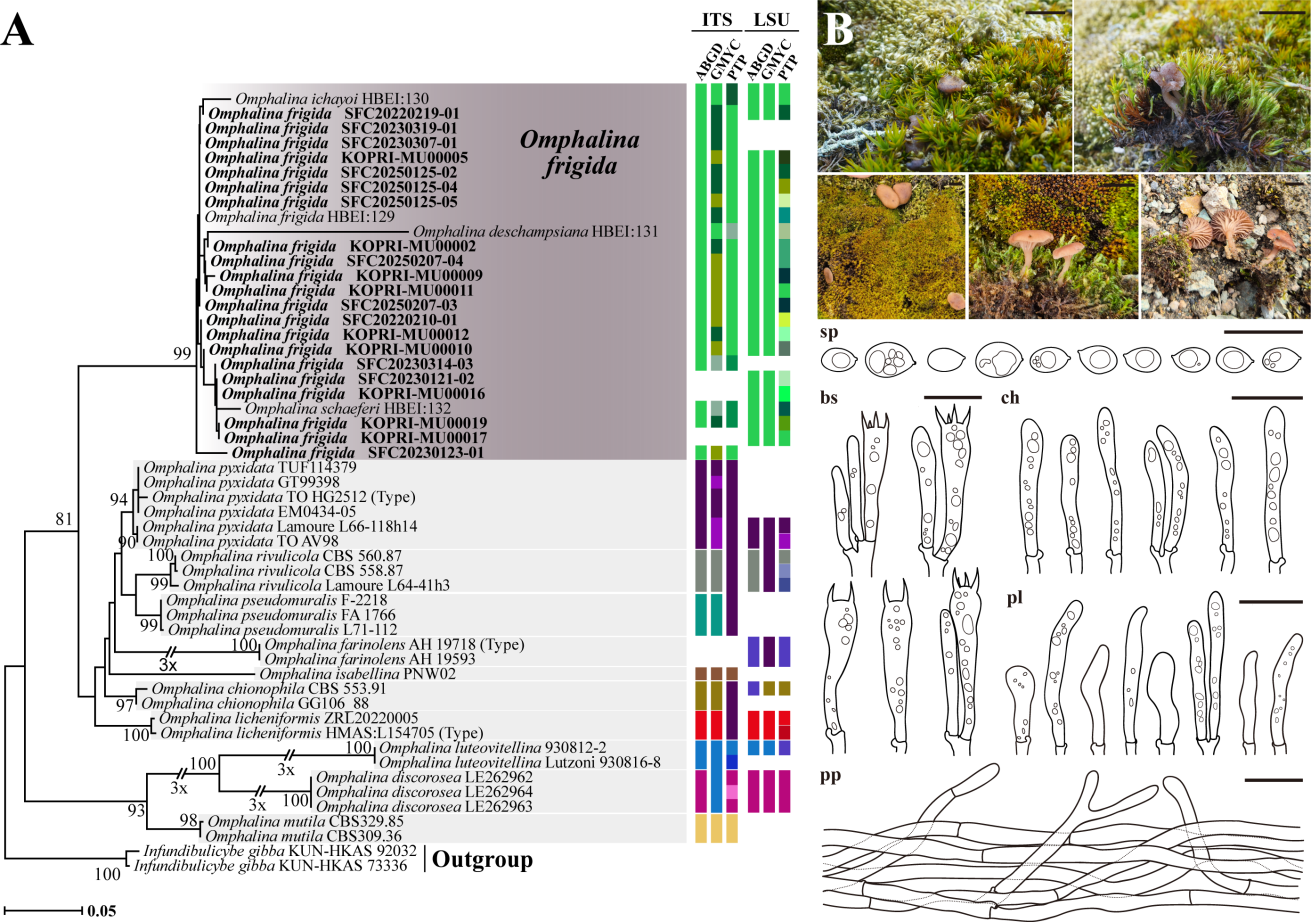
Phylogenetic tree, species delimitation results, and morphological characterization of Antarctic *Omphalina frigida*. **A**. Maximum likelihood phylogenetic tree of *Omphalina* based on concatenated ITS and LSU sequences. Colored bars adjacent to phylogeny represent putative species boundaries from ABGD, GMYC, and PTP analyses applied to ITS and LSU datasets, respectively. Bootstrap support values over 70 are shown at nodes; **B**. Morphological characters of Antarctic *Omphalina frigida* with field photographs of basidiomata and line drawings of microscopic structures. Abbreviations are as follows: sp = basidiospores, bs = basidia, ch = cheilocystidia, pl = pleurocystidia, pp = pileipellis. Scale bars: 1 cm (basidiomata); 20 μm (bs, ch, pl, pp, sp).

From a total of 51 specimens (50 Antarctic specimens and one Korean specimen), 47 ITS, 40 LSU, and 43 *RPB2* sequences were obtained. Phylogenetic analyses for *Arrhenia*, *Galerina*, and *Omphalina* were performed using concatenated datasets; ITS and LSU sequences were used for *Arrhenia* (Fig. 1A), whereas ITS, LSU, and *RPB2* sequences were used for *Galerina* (Fig. 2), and ITS and LSU sequences were used for *Omphalina* (Fig. 3A).

Within *Arrhenia*, all Antarctic specimens formed a single, well-supported clade (Bootstrap (BS) = 98), which includes only one public sequence labeled as “*A. antarctica*” (accession number PP151300). This clade was distinct from all described species. In *Omphalina*, Antarctic specimens grouped within a well-supported monophyletic clade (BS = 99) alongside *O. deschampsiana*, *O. frigida*, *O. ichayoi*, and *O. schaeferi*. For *Galerina*, Antarctic specimens formed two well-supported clades: one corresponding to *G. fallax* (BS = 98) and another including both *G. venenata* and *G. pseudomycenopsis* (BS = 70).

Species delimitation analyses (ABGD, GMYC, and PTP where applicable) were performed for all three genera, with results presented alongside RAxML phylogenetic trees (Figs 1A, 2, 3A). These analyses revealed varying tendencies compared to previously described species. In *Arrhenia*, among 41 described species, ABGD and GMYC each delimited 20 species, while PTP delimited 32 species, demonstrating moderate lumping and aligning most closely with prior taxonomic studies by grouping some debated species (*A. fusconigra*, *A. telmatiaea*, *A. umbritilis*, *A. svalbardensis*, and *A. acerosa*) (Fig. 1A). All analyses consistently recognized Antarctic *Arrhenia* specimens as a single, well-supported lineage distinct from all described species.

For *Galerina*, against 57 described species, ITS-based PTP delimited 64 species, closely matching the established species boundaries (Fig. 2A). GMYC delimited 65 species, indicating a slight tendency toward over-splitting, while ABGD excessively lumped all species into a single group. LSU-based analyses also exhibited excessive lumping, with ABGD delimiting two species, GMYC six species, and PTP 20 species (Fig. 2B). However, *RPB2*-based analyses produced the most consistent results, as all three methods delimited 10 species, reflecting moderate lumping and greater alignment with established taxonomic criteria. All LSU-based and *RPB2*-based methods consistently recognized that *G. pseudomycenopsis*, *G. venenata*, and our Antarctic specimens are conspecific.

For *Omphalina*, compared to 13 described species, ITS-based ABGD grouped these into 10 species, PTP delimited 11 species with minor lumping, and GMYC delimited 18 species but with inconsistencies in certain groupings (such as *O. pyxidata* clades, and the clade containing *O. discorosea* and *O. luteovitellina*) (Fig. 3A). In LSU-based analyses, ABGD delimited seven species, GMYC delimited four species, and PTP delimited 27 species, the highest number of species delimited across all analyses. The four Antarctic species (*O. deschampsiana*, *O. frigida*, *O. ichayoi*, and *O. schaeferi*), along with our Antarctic *Omphalina* specimens, were delimited as a single species by ITS-based ABGD, LSU-based ABGD, and LSU-based GMYC. In contrast, both ITS-based GMYC and ITS-based PTP over-split the Antarctic clade into four species, while LSU-based PTP delimited 16 species.

### Analysis of amanitin biosynthesis in Antarctic Galerina specimens

**Figure 4.**
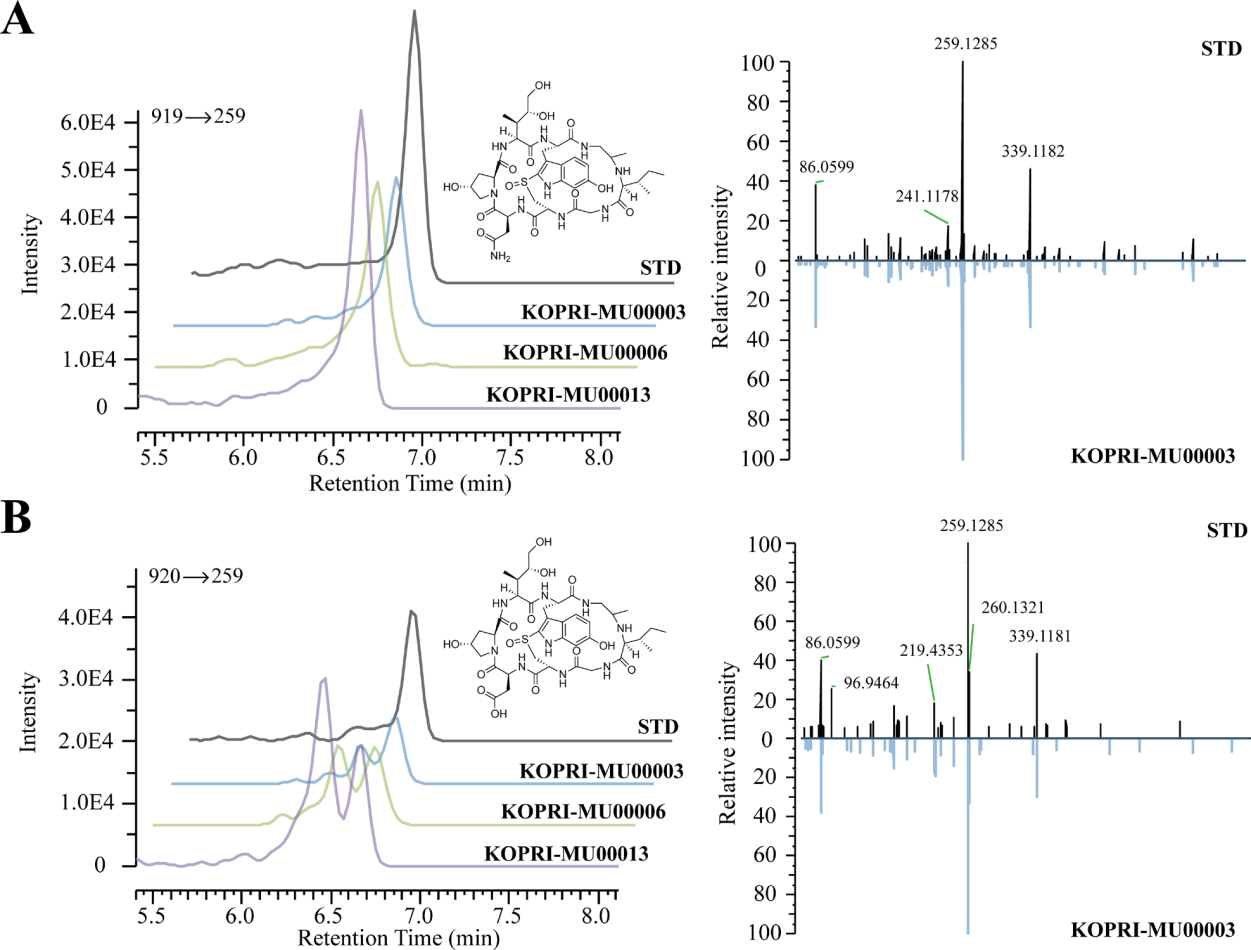
LC-MS/MS analysis of amanitins in Antarctic *Galerina venenata*. Chromatograms, chemical structures, and ion spectra of **A**. α-amanitin detected from three Antarctic *Galerina venenata* specimens; **B**. β-amanitin detected from the same three specimens.

LC-MS/MS analysis identified both α- and β-amanitin in all analysed samples (Table 2). The calibration curve for α-amanitin showed strong linearity (y = 2E-06x + 0.1873, R^2^ = 0.9993) across the dynamic ranges. The ethanol-preservation solution (1 ml absolute ethyl alcohol used to preserve approximately 0.5 cm^2^ of fresh tissue) from three Antarctic *Galerina* specimens contained 0.63 to 1.08 ppm α-amanitin (Fig. 4, Table 2). β-amanitin was also detected in all samples, with peak areas ranging from 115,909 to 432,965. However, absolute quantification was only performed for α-amanitin due to the availability of a calibrated standard, so only relative quantification was performed for β-amanitin. Relative peak intensities and α-amanitin concentration for each specimen are presented in Table 2.

**Table 2.** LC-MS/MS analysis of amanitin content in Antarctic *Galerina venenata* specimens. Standards of α-amanitin (10 ppm, 1 ppm, 100 ppb) were used to establish a calibration curve for α-amanitin quantification. Peak areas for both α- and β-amanitin are shown for three Antarctic *Galerina venenata* specimens, with calculated α-amanitin concentrations for each specimen.

**Sample**	**Peak Area**		**Concentration (ppm)**
	**β-amanitin**	**α-amanitin**	**α-amanitin**
Standard_100 ppb	–	28695.91	0.1
Standard_1 ppm		398055.69	1
Standard_10 ppm		5852610.23	10
**KOPRI-MU00003**	115909.39	266144.16	0.63396868
**KOPRI-MU00006**	226697.31	425173.08	0.900850959
**KOPRI-MU00013**	432965.25	530156.57	1.077034224

### Divergence-time estimation of Antarctic mushrooms

**Figure 5.**
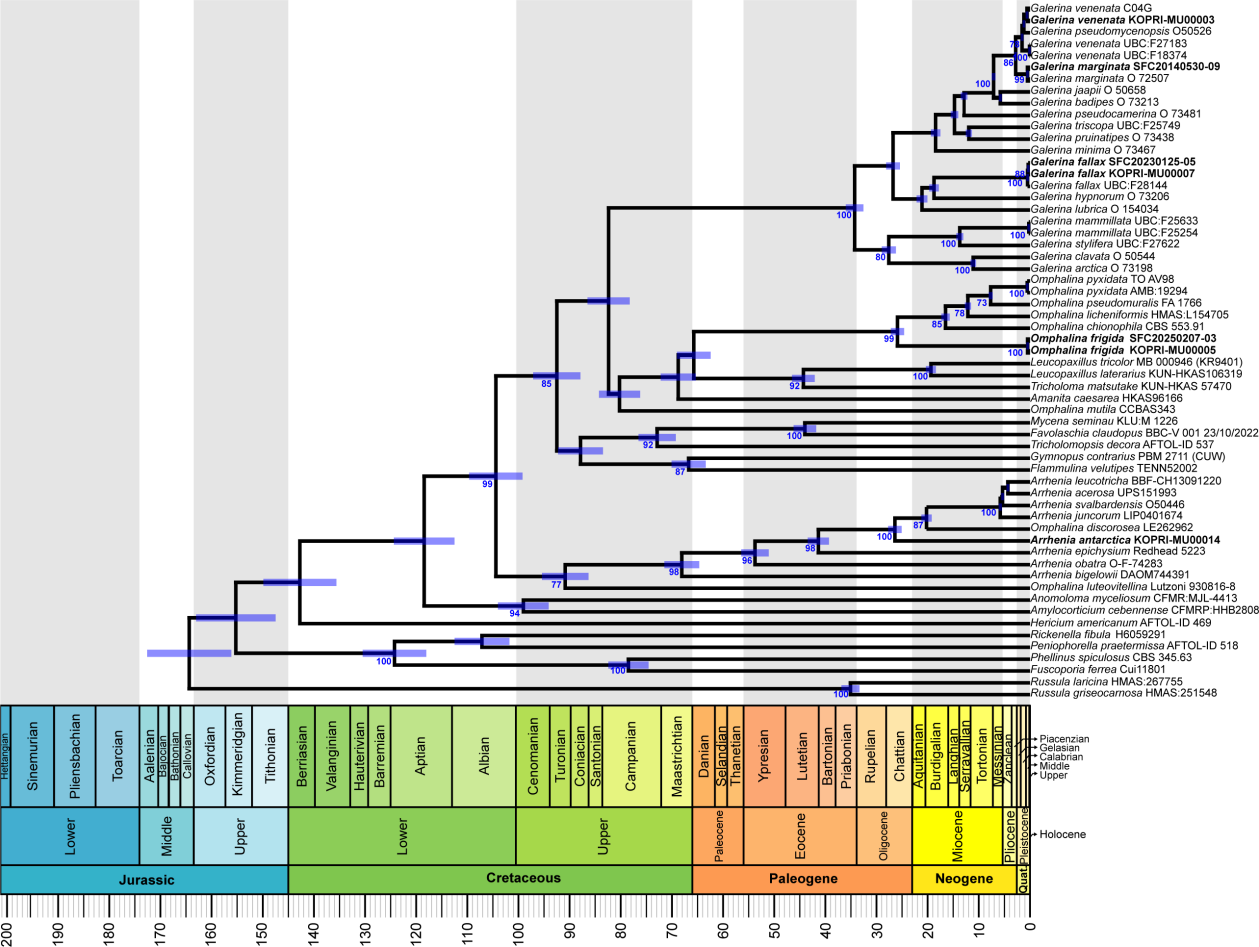
Divergence time estimation of Antarctic mushrooms based on multilocus dataset. Specimens sequenced in this study are indicated in bold. Mean divergence times (Mya) and bootstrap support values are shown at internodes. Horizontal blue bars represent 95% Highest Posterior Density (HPD) interval for divergence time estimates. The geological timescale is shown at the bottom.

The combined dataset (ITS1-5.8S-ITS2-LSU-*RPB2*) for the molecular dating included 59 specimens representing 50 taxa from *Agaricales*, *Amylocorticiales*, *Hymenochaetales*, and *Russulales* (Fig. 5). Divergence-time estimates indicated that the closest ancestor of Antarctic *Arrhenia* lineage diverged in the late Oligocene, with a mean stem age of 26.4 Mya and a 95% HPD of 15.38–31.48 Mya. The Antarctic *Omphalina* lineage showed a more recent divergence, with a mean stem age of 0.45 Mya (95% HPD: 0.08–0.944 Mya), corresponding to the Pleistocene. Two Antarctic *Galerina* species are also estimated to have diverged during the Pleistocene. Antarctic *G. fallax* was estimated to have diverged during the middle Pleistocene with a mean stem age of 0.46 Mya (95% HPD: 0.03–0.75 Mya), while Antarctic *G. venenata* was estimated slightly earlier than *G. fallax*, with a mean stem age of 0.57 Mya with a 95% HPD of 0.01–1.42 Mya.

### Taxonomy

#### Arrhenia antarctica

Taxon classificationFungiAgaricalesHygrophoraceae

(Singer) Redhead, Lutzoni, Moncalvo & Vilgalys, Mycotaxon 83: 46 (2002)

46987571-564F-57DD-B7F8-A9EBDD774E39

Fig. 1B

##### Description.

***Pileus*** 4–60 mm diameter, at first convex, becoming applanate, with slightly depressed (umbo) at the center, with a flaring margin, surface glabrous, hygrophanous, light brown (7D8) to reddish brown (8E3) when young in moisture, yellowish brown (5F8) to dark brown (7F4) when mature in moisture, orange gray (6B2) to brownish orange (6C4) or brownish gray (6C2) when dry. ***Lamellae*** subdistant to close, deeply decurrent, 12–47 lamellae with 1–5 lamellulae, irregularly forked, concolorous with the pileus. ***Stipe*** 4–37 × 2–7 mm, cylindric, concolorous with lamellae, central, solid. ***Context*** concolorous with stipe (but pallid).

***Basidiospores*** 5.5–9.5 × 4–6.5 μm, Lm × Wm = 7.39 × 5.02 μm, Q = 1.00–2.03, Qm = 1.45, ellipsoid, oblong to obovoid, some variation in shape. ***Basidia*** 25–35.6(–37.4) × 5.5–10.6 μm, mostly 4-spored with sometimes 2-spored, cylindrical to clavate. ***Cystidia*** absent. ***Pileipellis*** a cutis of parallel and thin-walled hyphae, 3.0–15.3 μm wide, hyaline to brownish, with incrusted brown pigment. ***Stipipellis*** a cutis of parallel and thin-walled hyphae, 3.2–17.6 μm wide, hyaline to brownish, substantially with fine incrusted brown pigment. ***Clamp connections*** present in all tissues.

##### Ecology.

Solitary, gregarious or caespitose on various moss (such as *Bryum pseudotriquetrum*, *Sanionia uncinata*, and *Syntrichia filaris*), often attached to living moss (*Sanionia uncinata*) with white mycelial tomentum, most abundant in late January or February in Antarctica.

##### Specimens examined.

See Suppl. material 1.

##### Notes.

This species was first described from Antarctica by Singer (1956) and has since been repeatedly recorded from vegetation covered regions of the continent, including King George Island (Bertazzo-Silva and Putzke 2025; Bertazzo-Silva et al. 2025c). Previous studies have demonstrated that this species exhibits high morphological plasticity in size and color, and substrate association (grasses, mosses, lichens, etc.) (Bertazzo-Silva et al. 2025c). This diversity highlights the species’ adaptability to the harsh environmental conditions of Antarctica. Newly collected specimens in this study indicate that the lamellae are concolorous with the pileus, without becoming noticeably pallescent, and all other characters closely correspond to Singer’s original description (Singer 1956).

#### Galerina fallax

Taxon classificationFungiAgaricalesHymenogastraceae

A.H. Sm. & Singer, Mycologia 47: 561 (1955)

33CF0304-D8FE-589E-8E50-9C709F22D47E

Fig. 6

##### Description.

***Pileus*** 3–20 mm diam, at first conic, hemispherical to spherical, becoming convex, with a flaring margin, surface glabrous, hygrophanous, reddish orange (7A8) to reddish orange (7C8) when moisture, fading to light brown (6D8) to light brown (7D8) in drying, crenate, and at first the disc with translucent striate. ***Lamellae*** distant to subdistant, decurrent, almost concolorous with pileus, a little bit lighter, grayish orange (6B4) to deep orange (6A8) when moisture, light orange (6A5) to orange (6A6). ***Stipe*** 3–30 × 1–5 mm, cylindrical or tapering upwards, brownish orange (6C6) to brownish orange (6C8) when moisture, light orange (6A4) to orange (6A6) when dry, entirely fibrillose with white fibrils. ***Veil*** not developed.

**Figure 6.**
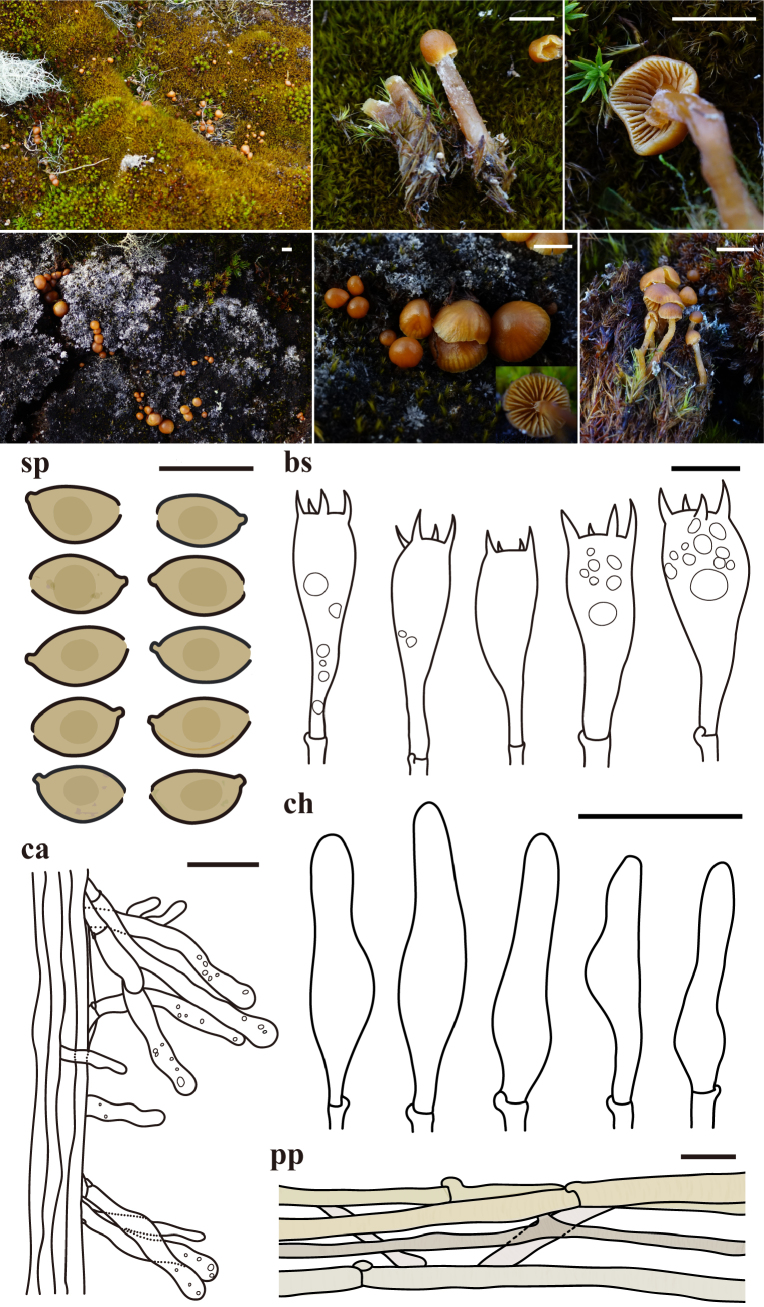
Morphological characters of Antarctic *Galerina fallax*. The top section presents field photographs of the basidiomata, while the bottom section presents line drawings of microscopic structures. Abbreviations are as follows: sp = basidiospores, bs = basidia, ch = cheilocystidia, ca = caulocystidia, pp = pileipellis. Scale bars: 1 cm (basidiomata), 10 μm (sp); 20 μm (bs, ca, ch, pp).

***Basidiospores*** (7.9–)8.3–10(–10.3) × (5.3–)5.5–6.3(–6.6) μm, Lm × Wm = 9.17 × 5.87 μm, Q = 1.4–1.76, Qm = 1.57, amygdaliform with acute apex in side view, ovoid in frontal view, thick-walled, yellowish-brown, with one large oil drop. ***Basidia*** (26–)28–34(–36) × 8–10 μm, (narrowly) clavate, 4–spored, hyaline, sometimes yellowish-brown in KOH. ***Pleurocystidia*** absent. ***Cheilocystidia*** 25.3–41 × 6.8–9.3 μm, (narrowly) lageniform to urticiform, with obtuse apex, thin-walled, hyaline in KOH. ***Pileipellis*** a cutis, terminal hyphae 5–11.5 μm wide, intertwined, thin-walled, hyaline in KOH, with yellowish brown incrusting pigment. ***Caulocystidia*** 18.4–58.8 × 4–9.1 μm, cylindrical to clavate, with subcapitate apex, mostly at the apex of the stipe, thin-walled, hyaline in KOH. ***Stipitipellis*** a cutis of parallel and thin-walled hyphae, 3–11.6 μm wide, hyaline in KOH. ***Clamp connections*** present in all tissues.

##### Ecology.

Gregarious on moss. The basidiomata were found to emerge from living portions of the moss tissue, especially associated with brownish part of the mat.

##### Specimens examined.

See Suppl. material 1.

##### Notes.

Compared with the original description of *G. fallax*, Antarctic specimens of *G. fallax* exhibit a slightly larger pileus (up to 20 mm vs. 5–10(–15) mm), larger basidia (28–34 × 8–10 μm vs. 17–20 × 6–7 μm), and cheilocystidia with a broader size range (25–41 × 6.8–9.3 μm vs. 24–35 × 6–9 μm) (Smith and Singer 1964; Groves 1965). Additionally, algal cells were observed entangled around the stipitipellis, and moss tissue was frequently found adhered to the basidiomata (data not shown). Putzke and Pereira (1996) proposed that *G. fallax* and *G. perrara* (proposed as a new species with Antarctic specimens) are conspecific and differ only in pileus size (<5 mm in the latter) (Putzke and Pereira 1996). Our observations support this interpretation, although Antarctic specimens consistently have a larger pileus.

#### Galerina venenata

Taxon classificationFungiAgaricalesHymenogastraceae

 A.H. Sm., Mycologia 45: 922 (1953)

C75F36AE-FFD2-5300-8DA8-7985E9A1563A

Fig. 7

##### Synonym.

*Galerina pseudomycenopsis* Pilát, Friesia 5 (1): 19 (1954).

##### Description.

***Pileus*** 2.5–30 mm diam, at first hemispherical, convex to broadly conical, finally expanding to applanate, with slightly depressed (umbo) at the center, surface glabrous, hygrophanous brownish orange (7C8) to brown (7E8) when moisture, orange (6A6) to light brown (6D8) when dry, crenate. ***Lamellae*** distant, broadly adnexed, grayish orange (6B3) to pastel red (7A4) when moisture, light orange (6A4) to brownish orange (6C4) when dry. ***Stipe*** 3–52 × 1.5–5 mm, cylindrical, central, white (7A1) to gray (7B1) when moist, orange-white (6A2) to light orange (6A4) when dry, entirely fibrillose with white fibrils, with a white basal mycelium. ***Veil*** well developed in mature specimens.

**Figure 7.**
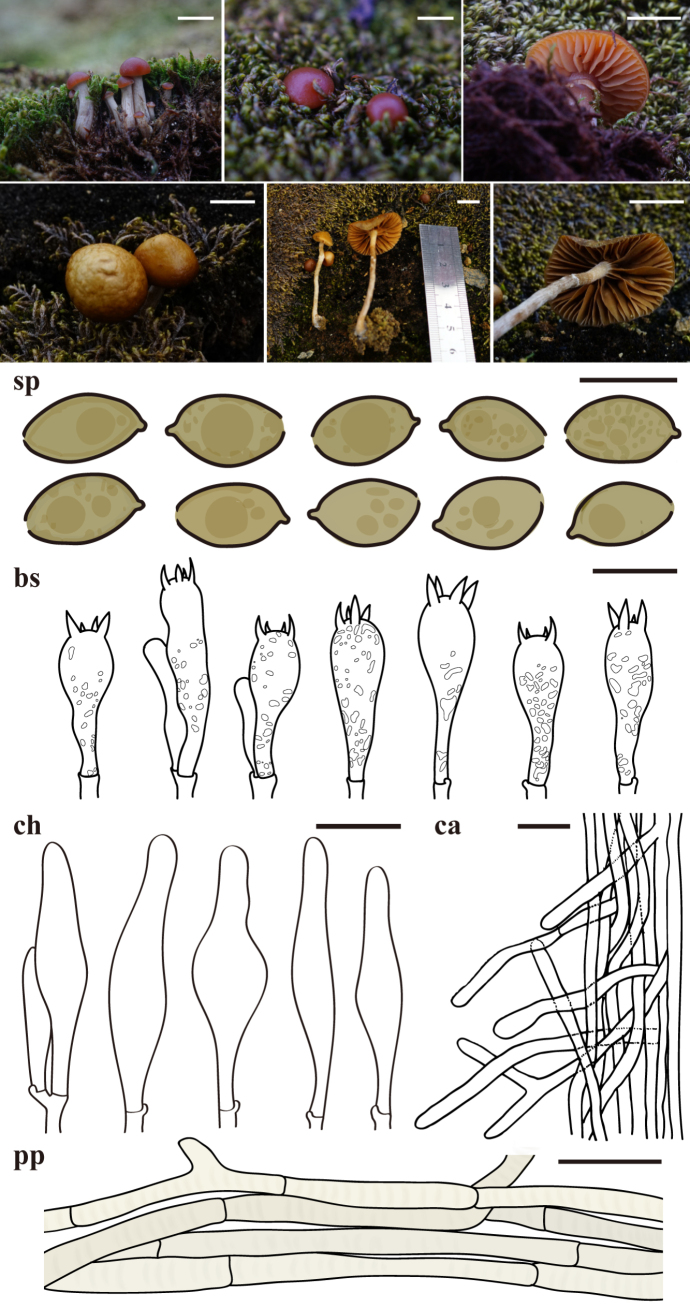
Morphological characters of Antarctic *Galerina venenata*. The top section presents field photographs of the basidiomata, while the bottom section presents line drawings of microscopic structures. Abbreviations are as follows: sp = basidiospores, bs = basidia, ch = cheilocystidia, ca = caulocystidia, pp = pileipellis. Scale bars: 1 cm (basidiomata); 10 μm (sp); 20 μm (bs, ca, ch, pp).

***Basidiospores*** (7.5–)8.5–11(–12) × 5.3–6.5(–7.0) μm, Lm × Wm = 9.99 × 6.14 μm, Q = 1.4–1.85, Qm = 1.62, amygdaliform with acute apex in side view, ovoid in frontal view, thick-walled, yellowish-brown, with oil drops. ***Basidia*** (26.7–)29–40(–43.5) × 7.8–13.0(–13.7) μm, clavate, 4–spored, hyaline. ***Cheilocystidia*** 36.5–74.5 × 7.5–18 μm, lageniform, utriform to fusiform, thin-walled. ***Pileipellis*** a cutis, 2–15.3 μm wide, thin-walled, hyaline to brownish, with fine incrusted brown pigment. ***Caulocystidia*** 26–98 × 4.5–14.2 μm, clavate to fusiform, mostly at the apex of the stipe, thin-walled. ***Stipitipellis*** a cutis of parallel and thin-walled hyphae, 2–9.3 μm wide. ***Clamp connections*** present in all tissues.

##### Ecology.

Gregarious, occasionally caespitose, on moss.

##### Specimens examined.

See Suppl. material 1.

##### Notes.

Phylogenetic analyses consistently classified the Antarctic specimens within the clade comprising *G. marginata*, *G. venenata*, and *G. pseudomycenopsis*, three taxa historically distinguished by their toxin profiles and ecological characters (Groves 1965; Gulden et al. 2005; Landry et al. 2021). Historically, *G. marginata* and *G. venenata* were distinguished by their toxin production (Groves 1965; Gulden et al. 2005; Landry et al. 2021), while *G. pseudomycenopsis* was distinguished based on ecological characters, particularly its preference for cold climates (Groves 1965; Gulden 1987; Gulden et al. 2005; Gulden 2010). However, toxin production and climate preference likely reflect physiological responses rather than taxonomic boundaries (Enjalbert et al. 2004; Akata et al. 2020), indicating that these criteria do not reliably delimit species. Considering all polyphasic evidence, including phylogenetic inference, morphological characteristics, and amanitin-producing characteristics, our Antarctic specimens are most appropriately assigned to *G. venenata*. The only notable morphological difference is the presence of slightly larger basidia (20–27 × 7–10 μm) (Groves 1965; Çelik et al. 2024), which suggests intraspecific morphological variation associated with environmental conditions.

#### Omphalina frigida

Taxon classificationFungiPoalesPoaceae

 Bertazzo-Silva, A.L. Costa & J. Putzke, Mycol. Progr. 24 (no. 41): 10 (2025)

CD95814A-72ED-5687-9B3A-A0C22EAADEC7

Fig. 3B

##### Synonyms.

*Omphalina deschampsiana* Bertazzo-Silva, A.L. Costa & J. Putzke, Mycol. Progr. 24 (no. 41): 5 (2025).

*Omphalina ichayoi* Bertazzo-Silva, A.L. Costa & J. Putzke, Mycol. Progr. 24 (no. 41): 8 (2025).

*Omphalina schaeferi* Bertazzo-Silva, A.L. Costa & J. Putzke, Mycol. Progr. 24 (no. 41): 12 (2025).

##### Description.

***Pileus*** 2–40 mm, at first convex to broadly conical, finally expanding to manifest slightly depressed (umbo) at the center, radially ribbed over the gills, hygrophanous, brownish gray (8E2) to grayish brown (9E3) when young in moisture, grayish brown (8D3) to grayish brown (8E3) when mature in moisture, reddish orange (7A6) to grayish red (7B5) when young in dry, light brown (7D5) in margin and reddish white (7A2) in center when mature in dry, finally becoming brownish gray with dark brown radial bands alternate over the gills (5C2). ***Lamellae*** distant to subdistant, decurrent, orange-gray (5B2) to orange-white (6A2) when in moisture, brownish orange (5B3) when dry. ***Stipe*** 2–20 × 0.5–5 mm, cylindrical, becoming glabrous, base covered by white tomentum, white (6A1) when young in moisture, white (6A1) to orange-gray (6B2) when young in dry, white (6A1), pale orange (6A3) to orange-gray (6B2) when mature.

***Basidiospores*** 6.5–9 × 4.5–6.2 µm, Q = 1.05–1.53, Qm = 1.3, amygdaliform in sideview, largely ellipsoid to obovoid in frontal view, smooth, thin-walled, hyaline, with some guttules. ***Basidia*** 34–49 × 7.5–10.5 µm, clavate, 2 or 4-spored. ***Cheilocystidia*** 28.8–46.6 × 3.8–9 µm, cylindrical, apically tapered, hyaline, thin-walled. ***Pleurocystidia*** 24–54.5 × 4.5–8.4 µm, similar to cheilocystidia. ***Pileipellis*** a cutis consisting of cylindrical, interwoven, 4–12 µm wide hyphae. ***Stipitipellis*** a cutis of parallel and thin-walled hyphae, 3.5–15 μm wide, hyaline in KOH. ***Caulocystidia*** 27.3–102.8 × 5–13.3 μm, cylindrical to clavate, hyaline in KOH. ***Clamp connections*** present in all tissues.

##### Ecology.

Gregarious, occasionally caespitose, on moss.

##### Specimens examined.

See Suppl. material 1.

##### Notes.

Both phylogenetic and species-delimitation analyses indicate that the four previously described Antarctic species—*O. deschampsiana*, *O. frigida*, *O. ichayoi*, and *O. schaeferi*—along with our newly collected specimens, form a single monophyletic lineage. No consistent morphological differences distinguishing these species were observed, and phylogenetic inference further supports their conspecificity. Given the low biodiversity of Antarctica and the probability that these lineages represent extant rather than recently diverged taxa (Fig. 5), all previously described Antarctic species and our newly collected specimens are treated as conspecific with *O. frigida*, whose epithet best reflects the ecological characteristics of this species, as the remaining epithets either honour individuals or reference *Deschampsia antarctica*, a host association not exclusive to this species (Bertazzo-Silva et al. 2025a). Although the geographic distribution, macromorphological characters, and ecological characters of this taxon also correspond to the original description of *O. ballesteri* (Calonge and Tomo 1991), direct comparison was not possible; this potential synonymy is therefore noted tentatively.

### Taxonomic Key to the Antarctic mushrooms of King George Island

**Table:** 

1	Pileus smooth, not radially ribbed; surface hygrophanous, brown to orange-brown or reddish orange; stipe usually fibrillose, without conspicuous basal tomentum	**2**
–	Pileus with distinct radial ribs or grooves when moist, often showing alternating darker bands when dry; color grayish brown to reddish orange; stipe slender, white to pale orange-gray with white basal tomentum	***Omphalina frigida***
2	Lamellae not deeply decurrent, or only slightly so; pileus smaller (< 30 mm), reddish orange to brownish orange; stipe cylindrical, often fibrillose with white fibrils	**3**
–	Lamellae deeply decurrent, concolorous with pileus (light to dark brown), not paling toward the margin; basidiomata solitary to gregarious on living *Sanionia* mats with white mycelial tomentum; pileus convex to applanate, margin flaring, 4–60 mm diam; stipe light brown, solid	***Arrhenia antarctica***
3	Veil absent, no ring zone; pileus smaller (3–20 mm), reddish orange when moist, fading to light brown when dry; lamellae clearly decurrent; stipe orange to brownish orange, entirely fibrillose, without ring	***G. fallax***
–	Partial veil distinct, forming a ring zone on the stipe; pileus 2.5–30 mm, brownish orange to brown, convex to applanate; lamellae broadly adnexed (not decurrent); stipe pale orange to brownish, with white fibrils and a clear ring zone	***G. venenata***

## Discussion

This study provides a comprehensive polyphasic taxonomic reassessment of mushrooms from the rapidly greening, ice-free regions surrounding Maxwell Bay, based on an integrated analysis of morphology, multilocus phylogenetics, species delimitation analyses, and chemical profiling. Through this polyphasic framework, it is demonstrated that Antarctic mushroom materials previously assigned to multiple names correspond to four species: *Arrhenia antarctica*, *Galerina fallax*, *Galerina venenata*, and *Omphalina frigida*. By refining species concepts in *Arrhenia*, *Omphalina*, and *Galerina*, we provide DNA sequence data, detailed morphological descriptions, and taxonomic identification keys for distinguishing the four Antarctic species.

By combining sequence data (ITS and LSU) with detailed morphological assessment, we clarify the phylogenetic placement of Antarctic *Arrhenia* and resolve the long-standing ambiguity over *Omphalina* in Antarctic records. *Arrhenia* is the most frequently documented genus, with five taxa reported in Antarctica (Bertazzo-Silva and Putzke 2025), but most have been identified solely based on morphology, with slight morphological differences (Horak 1966; Gumińska et al. 1994; Gyusheva and Chipev 1999; Putzke et al. 2012; Palfner et al. 2020). The consistently low ITS variation observed among geographically separated populations (distribution across ice-free areas of KGI and Nelson Island) indicates that *A. antarctica* constitutes a single, geographically widespread lineage. To discover true *Arrhenia* diversity in Antarctica, additional molecular investigations of the specimens of previous reports will be needed. Establishing a stable species boundary not only resolves previous taxonomic confusion but also provides a more reliable baseline for interpreting historical records and future biodiversity studies in the region.

For *Omphalina*, morphological analyses, phylogenetic analyses, and most species delimitation analyses strongly support four recently proposed Antarctic *Omphalina* species (Bertazzo-Silva et al. 2025a) and our specimens as a single, well-supported lineage. The adoption of *O. frigida* as the accepted name for this species carries broader taxonomic implications, as it highlights the importance of ecologically meaningful epithets—particularly given that names referencing specific host associations, *O. deschampsiana*, may inadvertently misrepresent the ecological breadth of this taxon. Given that *O. ballesteri* represents the earlier described *Omphalina* from Antarctica and that the morphological and ecological characters of our specimens closely correspond to its original description (Calonge and Tomo 1991), this raises the possibility of conspecificity. Whether *O. ballesteri* is conspecific with this lineage, however, remains unresolved, as direct comparision with its type specimen and sequence data was not possible; further examination of these materials will be essential to resolve this potential synonymy. Recognizing these taxa as conspecific stabilizes species concepts in *Omphalina* and mitigates taxonomic inflation, a persistent issue in low-diversity areas such as Antarctica. In these environments, taxonomic over-splitting can artificially inflate estimates of endemism and regional biogeographic significance, leading to misinterpretation in macroecological analyses and confusion in conservation planning (Isaac et al. 2004). A well-known example is the case of Antarctic skuas, where taxa were historically classified as two distinct species (*Stercorarius maccormicki* and *S. antarctica lonnbergi*) based on geographic isolation, morphology, and feeding ecology, but extensive hybridization was later discovered, prompting debate over whether previous classifications reflect taxonomic inflation rather than true evolutionary independence (Ritz et al. 2006; Ritz et al. 2008; Mota et al. 2023). In the context of Antarctic *Omphalina*, our results caution against overestimating species diversity through premature splitting, highlighting the necessity for integrative evidence to prevent the inflation of biodiversity estimates that may not represent actual evolutionary divergence.

The integrated taxonomic analysis conducted in this study necessitates a revision of the taxonomic status of *Galerina* species. Many Antarctic *Galerina* materials have previously been identified as *G. marginata* or *G. pseudomycenopsis* (Gumińska et al. 1994; Garrido-Benavent et al. 2023; Bertazzo-Silva et al. 2024), taxa that are part of the problematic *G. marginata* species complex. Our morphological, multilocus phylogenetic, and chemical analyses consistently identified the Antarctic materials as *G. venenata*. *Galerina pseudomycenopsis* is morphologically and phylogenetically indistinguishable from *G. venenata*, having been historically distinguished based primarily on the ecological distribution (Gulden 1987, 2010; Khovpachev et al. 2022). To date, toxin production in *G. pseudomycenopsis* has not been investigated, contributing to ongoing taxonomic confusion. However, LC-MS/MS analysis confirmed the presence of α- and β-amanitin biosynthesis potential in this species, providing the first evidence of amanitin production in Antarctic *Galerina*. The combined morphological, phylogenetic, and chemical evidence supports synonymizing *G. pseudomycenopsis* with *G. venenata*. Furthermore, variations in amanitin concentrations and β/α-amanitin ratios are more plausibly explained by physiological or developmental differences rather than taxonomic divergence (Enjalbert et al. 2004; Luo et al. 2012). Therefore, it is unreliable to classify species based on the presence of the amanitin, since the ability to synthesize the substance may be a species trait, but the amount synthesized under observed conditions falls within the range of phenotypic plasticity. By clarifying these taxonomic boundaries, our reassessment defines Antarctic *Galerina* diversity and addresses a long-standing source of confusion within the *G. marginata* species complex, indicating that Antarctic records previously identified as members of the *G. marginata* species complex are more accurately interpreted as *G. venenata*.

With reliable species identities, divergence-time estimates reveal different evolutionary histories that closely correlate with major Antarctic environmental changes. Antarctic *Galerina* and *Omphalina* seem to have diversified during the Pleistocene, a period characterized by repeated glacial-interglacial cycles and intermittent expansion of Antarctic vegetations, suggesting recent colonization or re-expansion under favorable conditions (Fernández‐Mendoza and Printzen 2013; Pisa et al. 2014; Garrido-Benavent et al. 2021). The estimated divergence time for Antarctic *Galerina* in this study aligns with previous estimates for Antarctic *G. marginata* (Garrido-Benavent et al. 2023), which now corresponds to *G. venenata*. This concordance indicates that earlier divergence time estimates remain accurate, even as the lineage is now recognized as *G. venenata* under our revised taxonomy. In contrast, the closest ancestral lineage of *A. antarctica* was estimated to have diversified during the Oligocene, coinciding with early glaciation following continental isolation, and may have persisted through extended habitat loss (López-Quirós et al. 2021); however, limited taxon-sampling could also serve as an alternative explanation. Palynological and fossil records, including plant-bearing cherts or silicified wood with fossil fungal remnants (Harper et al. 2016; Amenábar et al. 2025) from KGI and adjacent archipelagos, indicate continuous presence of vegetation even during the Oligocene (Stuchlik 1981; Mohr 2001; Poole et al. 2001; López-Quirós et al. 2021; Bastias-Silva et al. 2025). Despite differing evolutionary estimates, all studied species have dark-pigmented basidiomata (Bertazzo-Silva et al. 2025c), a trait that helps them survive in the extreme Antarctic environment. Pigments such as melanin and carotenoids enhance membrane stability and stress resistance, indicating convergent adaptation (Jagannadham et al. 2000; Krah et al. 2019; Sajjad et al. 2020). Although evolutionary timelines differ, these findings demonstrate that Antarctic mushrooms comprise both old and relatively young lineages that have converged on shared adaptive traits to persist in the most challenging terrestrial ecosystems on Earth.

## Conclusions

This study represents the first polyphasic taxonomic reassessment of Antarctic mushrooms, resolving long-standing taxonomic ambiguities and establishing a foundation for understanding fungal diversity in the rapidly changing Antarctic environment. By clarifying species boundaries and addressing unsupported taxonomic inflation, our results provide a more reliable assessment of evolutionary patterns and biogeography among Antarctic mushrooms. The contrasting evolutionary origins observed among lineages, together with shared adaptive traits, highlight the capacity of fungi to survive through major environmental transitions. To further elucidate adaptive mechanisms and responses to ongoing climate change, population-level sampling with genomic approaches will be essential. As Antarctic ice-free areas expand, these findings will help researchers in monitoring microbial responses to climate change, utilizing mushrooms as microbial indicators of ecosystem change in polar regions.

## Supplementary Material

XML Treatment for Arrhenia antarctica

XML Treatment for Galerina fallax

XML Treatment for Galerina venenata

XML Treatment for Omphalina frigida

## Additional information

### Artificial Intelligence (AI) use

Regarding the use of AI in the preparation of this manuscript, the authors declare the following: Claude, GPT.

Used for: Language, style and writing.

### Author ORCIDs

J.S. Kim https://orcid.org/0000-0003-1869-7347

J. Choi https://orcid.org/0009-0008-7737-6467

M.-S. Choi https://orcid.org/0000-0002-8053-3805

K.M. Kim https://orcid.org/0000-0001-5125-5079

Y. Joo https://orcid.org/0000-0001-8245-7693

J.R. Cabrera-Pardo https://orcid.org/0000-0001-6978-581X

Y.W. Lim https://orcid.org/0000-0003-2864-3449

### Data availability

All sequences generated in this study have been deposited in GenBank, and the corresponding accession numbers are provided in Table 1.

## Supplementary materials

Supplementary material 1 Collection information of specimens used in this study Data type xlsx https://binary.pensoft.net/file/1650063This dataset is made available under the Open Database License (http://opendatacommons.org/licenses/odbl/1.0/). The Open Database License (ODbL) is a license agreement intended to allow users to freely share, modify, and use this Dataset while maintaining this same freedom for others, provided that the original source and author(s) are credited. Ji Seon Kim, Jiyun Choi, Min-Soo Choi, Kyung Mo Kim, Jae Eun So, Youngsung Joo, Jaime R. Cabrera-Pardo, Young Woon Lim

Supplementary material 2 List of species and GenBank accession numbers of sequences used in phylogenetic analyses Data type xlsx https://binary.pensoft.net/file/1650064This dataset is made available under the Open Database License (http://opendatacommons.org/licenses/odbl/1.0/). The Open Database License (ODbL) is a license agreement intended to allow users to freely share, modify, and use this Dataset while maintaining this same freedom for others, provided that the original source and author(s) are credited. Ji Seon Kim, Jiyun Choi, Min-Soo Choi, Kyung Mo Kim, Jae Eun So, Youngsung Joo, Jaime R. Cabrera-Pardo, Young Woon Lim

## References

[B1] Abràmoff MD, Magalhães PJ, Ram SJ (2004) Image processing with ImageJ. Biophotonics international 11: 36–42. 10.1016/b978-1-4557-0737-9.00006-0

[B2] Akata I, Yilmaz I, Kaya E, Coskun NC, Donmez M (2020) Toxin components and toxicological importance of *Galerina marginata* from Turkey. Toxicon 187: 29–34. 10.1016/j.toxicon.2020.08.017 32866473

[B3] Amenábar CR, Guerstein GR, Casadío S, Alperín MI (2025) Palynological Evidence for a Late Eocene Age of the Submeseta Formation from Marambio Island, Antarctic Peninsula-Environmental Change Prior to the Onset of Major Antarctic Glaciation. Ameghiniana 62: 110–129. 10.5710/AMGH.28.02.2025.3621

[B4] Bambauer TP, Wagmann L, Weber AA, Meyer MR (2020) Analysis of α-and β-amanitin in human plasma at subnanogram per milliliter levels by reversed phase ultra-high performance liquid chromatography coupled to orbitrap mass spectrometry. Toxins 12: 671. 10.3390/toxins12110671 PMC7690657 33113909

[B5] Barreda VD, Palazzesi L, Olivero EB (2019) When flowering plants ruled Antarctica: evidence from Cretaceous pollen grains. New Phytologist 223: 1023–1030. 10.1111/nph.15823 30924945

[B6] Bastias-Silva J, Leppe M, Manriquez L, Trevisan C, Fox BR, Magiera M, Wilson G, Tavazzani L, Chelle-Michou C, Gao L (2025) Neogene plant macrofossils from West Antarctica reveal persistence of Nothofagaceae forests into the early Miocene. Communications Earth & Environment 6: 965. 10.1038/s43247-025-02921-x PMC12657238 41323747

[B7] Bell MA, Lloyd GT (2015) strap: an R package for plotting phylogenies against stratigraphy and assessing their stratigraphic congruence. Palaeontology 58: 379–389. 10.1111/pala.12142

[B8] Bengtsson‐Palme J, Ryberg M, Hartmann M, Branco S, Wang Z, Godhe A, De Wit P, Sánchez‐García M, Ebersberger I, de Sousa F (2013) Improved software detection and extraction of ITS1 and ITS 2 from ribosomal ITS sequences of fungi and other eukaryotes for analysis of environmental sequencing data. Methods in Ecology and Evolution 4: 914–919. 10.1111/2041-210X.12073

[B9] Bertazzo-Silva FA, Costa AL, Furlan-Lopes C, D’Ávila MF, Carvalho EL, Putzke J, Schaefer CEGR (2025a) Cold discoveries: morphological and phylogenetic analyses unveil four new species of *Omphalina* (*Agaricales*, *Basidiomycota*) in Antarctica. Mycological Progress 24: 1–17. 10.1007/s11557-025-02063-6

[B10] Bertazzo-Silva FA, Costa AL, Velloso JRP, Sousa FHA, Schaefer CEGR, Putzke J (2025b) Exploratory interaction of *Chionis alba* (snowy sheathbill) with the amatoxin-producing mushroom *Galerina marginata* in Antarctica. Infection, Genetics and Evolution 105822. 10.1016/j.meegid.2025.105822 40945877

[B11] Bertazzo-Silva FA, Putzke J (2025) *Agaricales* from Antarctica: Diversity of basidiomata, research challenges, and future perspectives in polar environments. Fungal Biology Reviews 54: 100458. 10.1016/j.fbr.2025.100458

[B12] Bertazzo-Silva FA, Putzke J, Furlan-Lopes C, D’ávila MF, Costa AL, Carvalho EL, Zorzi AF, Schaefer CEGR (2024) Expanding geographic distribution knowledge of *Galerina marginata* (Batsch) Kühner (*Agaricales*, *Hymenogastraceae*) with a novel Antarctic record. Biodiversity Data Journal 12: e125727. 10.3897/BDJ.12.e125727 PMC11214013 38948134

[B13] Bertazzo-Silva FA, Putzke J, Meira JL, Putzke MTL, Schaefer CEGR (2025c) Morphological Plasticity and Abundance Patterns of *Arrhenia antarctica* in the South Shetland Islands: Implications for Fungal Ecology in a Warming Antarctica. Diversity 17: 489. 10.3390/d17070489

[B14] Bouckaert R, Vaughan TG, Barido-Sottani J, Duchêne S, Fourment M, Gavryushkina A, Heled J, Jones G, Kühnert D, De Maio N (2019) BEAST 2.5: An advanced software platform for Bayesian evolutionary analysis. PLoS Computational Biology 15: e1006650. 10.1371/journal.pcbi.1006650 PMC6472827 30958812

[B15] Calonge FD, Tomo AP (1991) *Omphalina ballesteri* (*Basidiomycotina*), una especie nueva en la Antártica. Boletín de la Sociedad Micológica de Madrid 16: 47–52.

[B16] Çelik A, Türkekul İ, Kaygusuz O (2024) First record of the deadly poisonous *Galerina venenata* (*Hymenogastraceae*, *Agaricomycotina*) from Türkiye. Anatolian Journal of Botany 8: 34–38. 10.30616/ajb.1396300

[B17] Colesie C, Walshaw CV, Sancho LG, Davey MP, Gray A (2023) Antarctica’s vegetation in a changing climate. Wiley Interdisciplinary Reviews: Climate Change 14: e810. 10.1002/wcc.810

[B18] Drummond AJ, Ho SYW, Phillips MJ, Rambaut A (2006) Relaxed phylogenetics and dating with confidence. PLoS Biology 4: e88. 10.1371/journal.pbio.0040088 PMC1395354 16683862

[B19] Duran J, Rodriguez A, Heiðmarsson S, Lehmann JR, Del Moral Á, Garrido-Benavent I, De los Rios A (2021) Cryptogamic cover determines soil attributes and functioning in polar terrestrial ecosystems. Science of The Total Environment 762: 143169. 10.1016/j.scitotenv.2020.143169 33131854

[B20] Enjalbert F, Cassanas G, Rapior S, Renault C, Chaumont J-P (2004) Amatoxins in wood-rotting *Galerina marginata*. Mycologia 96: 720–729. 10.1080/15572536.2005.11832920 21148893

[B21] Fernández‐Mendoza F, Printzen C (2013) Pleistocene expansion of the bipolar lichen *Cetraria aculeata* into the Southern hemisphere. Molecular Ecology 22: 1961–1983. 10.1111/mec.12210 23402222

[B22] Francis J, Ashworth A, Cantrill D, Crame J, Howe J, Stephens R, Tosolini A, Thorn V (2008) 100 million years of Antarctic climate evolution: evidence from fossil plants. National Academies Press, Washington, DC. 10.3133/ofr20071047kp03

[B23] Fujisawa T, Barraclough TG (2013) Delimiting species using single-locus data and the Generalized Mixed Yule Coalescent approach: a revised method and evaluation on simulated data sets. Systematic Biology 62: 707–724. 10.1093/sysbio/syt033 PMC3739884 23681854

[B24] Gardes M, Bruns TD (1993) ITS primers with enhanced specificity for basidiomycetes‐application to the identification of mycorrhizae and rusts. Molecular Ecology 2: 113–118. 10.1111/j.1365-294X.1993.tb00005.x 8180733

[B25] Garrido-Benavent I, Blanchette RA, De Los Ríos A (2023) Deadly mushrooms of the genus *Galerina* found in Antarctica colonized the continent as early as the Pleistocene. Antarctic Science 35: 345–358. 10.1017/S0954102023000196

[B26] Garrido-Benavent I, Pérez-Ortega S, de Los Ríos A, Mayrhofer H, Fernández-Mendoza F (2021) Neogene speciation and Pleistocene expansion of the genus *Pseudephebe* (*Parmeliaceae*, lichenized fungi) involving multiple colonizations of Antarctica. Molecular Phylogenetics and Evolution 155: 107020. 10.1016/j.ympev.2020.107020 33242583

[B27] Green TA, Schroeter B, Sancho LG (2007) Plant life in Antarctica. In: Functional plant ecology. CRC press, 389–434. 10.1201/9781420007626-13

[B28] Groves JW (1965) A Monograph of the Genus *Galerina* Earle. Hafner Publishing Company: New York. 10.2307/3756754

[B29] Guglielmin M, Dalle Fratte M, Cannone N (2014) Permafrost warming and vegetation changes in continental Antarctica. Environmental Research Letters 9: 045001. 10.1088/1748-9326/9/4/045001

[B30] Gulden G (1987) The genus *Galerina* on Svalbard. In: Arctic and alpine mycology II. Springer, 177–204. 10.1007/978-1-4757-1939-0_13

[B31] Gulden G (2010) *Galerinas* in cold climates. North American Fungi 5: 127–137. 10.2509/naf2010.005.0058

[B32] Gulden G, Stensrud Ø, Shalchian-Tabrizi K, Kauserud Hv (2005) *Galerina* Earle: a polyphyletic genus in the consortium of dark-spored agarics. Mycologia 97: 823–837. 10.1080/15572536.2006.11832774 16457352

[B33] Gumińska B, Heinrich Z, Olech M (1994) Macromycetes of the South Shetland Islands (Antarctica). Polish Polar Research, 103–109.

[B34] Gyusheva M, Chipev N (1999) *Arrhenia salina* (Hoiland) Gulden - new macromycete species to Livingston Island, South Shetlands, Maritime Antarctic. Bulgarian Antarctic Research: Life Sciences 2: 26–32.

[B35] Harmon LJ, Weir JT, Brock CD, Glor RE, Challenger W (2008) GEIGER: investigating evolutionary radiations. Bioinformatics 24: 129–131. 10.1093/bioinformatics/btm538 18006550

[B36] Harper CJ, Taylor TN, Krings M, Taylor EL (2016) Structurally preserved fungi from Antarctica: diversity and interactions in late Palaeozoic and Mesozoic polar forest ecosystems. Antarctic Science 28: 153–173. 10.1017/S0954102016000018

[B37] Hill RS, Scriven LJ (1995) The angiosperm-dominated woody vegetation of Antarctica: a review. Review of Palaeobotany and Palynology 86: 175–198. 10.1016/0034-6667(94)00149-E

[B38] Horak E (1966) On two new species of mushrooms collected in the Antarctic. Contribucion del Instituto Antárctico Argentino 104: 1–13.

[B39] Isaac NJ, Mallet J, Mace GM (2004) Taxonomic inflation: its influence on macroecology and conservation. Trends in Ecology & Evolution 19: 464–469. 10.1016/j.tree.2004.06.004 16701308

[B40] Jagannadham MV, Chattopadhyay MK, Subbalakshmi C, Vairamani M, Narayanan K, Mohan Rao C, Shivaji S (2000) Carotenoids of an Antarctic psychrotolerant bacterium, *Sphingobacterium antarcticus*, and a mesophilic bacterium, *Sphingobacterium multivorum*. Archives of Microbiology 173: 418–424. 10.1007/s002030000163 10896223

[B41] Kapli P, Lutteropp S, Zhang J, Kobert K, Pavlidis P, Stamatakis A, Flouri T (2017) Multi-rate Poisson tree processes for single-locus species delimitation under maximum likelihood and Markov chain Monte Carlo. Bioinformatics 33: 1630–1638. 10.1093/bioinformatics/btx025 PMC5447239 28108445

[B42] Katoh K, Rozewicki J, Yamada KD (2019) MAFFT online service: multiple sequence alignment, interactive sequence choice and visualization. Briefings in Bioinformatics 20: 1160–1166. 10.1093/bib/bbx108 PMC6781576 28968734

[B43] Khovpachev AA, Kalinina LB, Bolshakov SY, Volobuev SV, Ivanov IM, Yudin MA, Basharin VA, Chepur SV (2022) Distribution of amanitine-containing macromycetes in the territory of Russia. Toksikologicheskiy vestnik (Toxicological Review) 30: 85–93. 10.47470/0869-7922-2022-30-2-85-93

[B44] Kim JH, Ahn I-Y, Lee KS, Chung H, Choi H-G (2007) Vegetation of Barton peninsula in the neighbourhood of king Sejong Station (King George Island, maritime Antarctic). Polar Biology 30: 903–916. 10.1007/s00300-006-0250-2

[B45] Klarenberg IJ, Liu R, Convey P, Cornelissen JH, Bokhorst S (2026) How the small host the small: cryptogam trait‐mediated structuring of Antarctic microarthropod communities. Ecography 2026: e08175. 10.1002/ecog.08175

[B46] Kornerup A, Wanscher JH (1967) Methuen handbook of colour.

[B47] Krah F-S, Büntgen U, Schaefer H, Müller J, Andrew C, Boddy L, Diez J, Egli S, Freckleton R, Gange AC (2019) European mushroom assemblages are darker in cold climates. Nature Communications 10: 2890. 10.1038/s41467-019-10767-z PMC6599080 31253790

[B48] Landry B (2019) Phylogenetic distribution of alpha-amanitin producing *Galerina* of British Columbia (Doctoral dissertation, University of British Columbia).

[B49] Landry B, Whitton J, Bazzicalupo AL, Ceska O, Berbee ML (2021) Phylogenetic analysis of the distribution of deadly amatoxins among the little brown mushrooms of the genus *Galerina*. PLoS ONE 16: e0246575. 10.1371/journal.pone.0246575 PMC7875387 33566818

[B50] Lee YI, Lim HS, Yoon HI (2009) Carbon and nitrogen isotope composition of vegetation on King George Island, maritime Antarctic. Polar Biology 32: 1607–1615. 10.1007/s00300-009-0659-5

[B51] Lepage T, Bryant D, Philippe H, Lartillot N (2007) A general comparison of relaxed molecular clock models. Molecular Biology and Evolution 24: 2669–2680. 10.1093/molbev/msm193 17890241

[B52] Longton R (1979) Vegetation ecology and classification in the Antarctic Zone. Canadian Journal of Botany 57: 2264–2278. 10.1139/b79-273

[B53] López-Quirós A, Escutia C, Etourneau J, Rodríguez-Tovar FJ, Roignant S, Lobo FJ, Thompson N, Bijl PK, Bohoyo F, Salzmann U (2021) Eocene-Oligocene paleoenvironmental changes in the South Orkney Microcontinent (Antarctica) linked to the opening of Powell Basin. Global and Planetary Change 204: 103581. 10.1016/j.gloplacha.2021.103581

[B54] Luo H, Hallen-Adams HE, Scott-Craig JS, Walton JD (2012) Ribosomal biosynthesis of α-amanitin in *Galerina marginata*. Fungal Genetics and Biology 49: 123–129. 10.1016/j.fgb.2011.12.005 PMC3997167 22202811

[B55] Matheny PB (2005) Improving phylogenetic inference of mushrooms with *RPB1* and *RPB2* nucleotide sequences (*Inocybe*; *Agaricales*). Molecular Phylogenetics and Evolution 35: 1–20. 10.1016/j.ympev.2004.11.014 15737578

[B56] McLoughlin S (2001) The breakup history of Gondwana and its impact on pre-Cenozoic floristic provincialism. Australian Journal of Botany 49: 271–300. 10.1071/BT00023

[B57] Mohr BA (2001) The development of Antarctic fern floras during the Tertiary, and palaeoclimatic and palaeobiogeographic implications. Palaeontographica Abteilung B, 167–208. 10.1127/palb/259/2001/167

[B58] Mota ACM, Costa ES, Torres JPM, de Araujo J, Tormena LC, Pires de Mendonça Dantas G (2023) Brown Skua and south polar Skua (*Aves*: *Stercorariidae*) a hybridization case or same species? Polar Biology 46: 1191–1201. 10.1007/s00300-023-03193-x

[B59] Palfner G, Binimelis-Salazar J, Alarcón ST, Torres-Mellado G, Gallegos G, Pea-Cortés F, Casanova-Katny A (2020) Do new records of macrofungi indicate warming of their habitats in terrestrial Antarctic ecosystems? Czech Polar Reports 10: 281–296. 10.5817/CPR2020-2-21

[B60] Paradis E, Schliep K (2019) ape 5.0: an environment for modern phylogenetics and evolutionary analyses in R. Bioinformatics 35: 526–528. 10.1093/bioinformatics/bty633 30016406

[B61] Parnikoza I, Miryuta N, Ozheredova I, Kozeretska I, Smykla J, Kunakh V, Convey P (2015) Comparative analysis of *Deschampsia antarctica* Desv. population adaptability in the natural environment of the Admiralty Bay region (King George Island, maritime Antarctic). Polar Biology 38: 1401–1411. 10.1007/s00300-015-1704-1

[B62] Pegler DN, Spooner B, Smith RL (1980) Higher fungi of Antarctica, the subantarctic zone and Falkland Islands. Kew Bulletin, 499–562. 10.2307/4110020

[B63] Pisa S, Biersma E, Convey P, Patiño J, Vanderpoorten A, Werner O, Ros RM (2014) The cosmopolitan moss *Bryum argenteum* in Antarctica: recent colonisation or in situ survival? Polar Biology 37: 1469–1477. 10.1007/s00300-014-1537-3

[B64] Poole I, Hunt RJ, Cantrill DJ (2001) A fossil wood flora from King George Island: ecological implications for an Antarctic Eocene vegetation. Annals of Botany 88: 33–54. 10.1006/anbo.2001.1425

[B65] Puillandre N, Lambert A, Brouillet S, Achaz G (2012) ABGD, Automatic Barcode Gap Discovery for primary species delimitation. Molecular Ecology 21: 1864–1877. 10.1111/j.1365-294X.2011.05239.x 21883587

[B66] Putzke J, Pereira A (1996) Macroscopic fungi from the South Shetland Islands, Antarctica. Revista Série Científica del INACH, Santiago-Chile 46: 31–39. 10.31789/pab.v3n1.010

[B67] Putzke J, Putzke MTL, Pereira AB, Albuquerque M (2012) *Agaricales (Basidiomycota)* fungi in the South Shetland Islands, Antarctica. INCT-APA Annual Activity Report, 71–74. 10.4322/APA.2014.065

[B68] Rambaut A, Drummond AJ, Xie D, Baele G, Suchard MA (2018) Posterior summarization in Bayesian phylogenetics using Tracer 1.7. Systematic Biology 67: 901–904. 10.1093/sysbio/syy032 PMC6101584 29718447

[B69] Redhead SA, Lutzoni F, Moncalvo JM, Vilgalys R (2002) Phylogeny of agarics: partial systematics solutions for core omphalinoid genera in the *Agaricales* (euagarics). Mycotaxon 83: 19–57. 10.5962/p.414960

[B70] Ritz MS, Hahn S, Janicke T, Peter H-U (2006) Hybridisation between South polar skua (*Catharacta maccormicki*) and Brown skua (*C. antarctica lonnbergi*) in the Antarctic Peninsula region. Polar Biology 29: 153–159. 10.1007/s00300-005-0034-0

[B71] Ritz MS, Millar C, Miller GD, Phillips RA, Ryan P, Sternkopf V, Liebers-Helbig D, Peter H-U (2008) Phylogeography of the southern skua complex—rapid colonization of the southern hemisphere during a glacial period and reticulate evolution. Molecular Phylogenetics and Evolution 49: 292–303. 10.1016/j.ympev.2008.07.014 18706509

[B72] Roland TP, Bartlett OT, Charman DJ, Anderson K, Hodgson DA, Amesbury MJ, Maclean I, Fretwell PT, Fleming A (2024) Sustained greening of the Antarctic Peninsula observed from satellites. Nature Geoscience 17: 1121–1126. 10.1038/s41561-024-01564-5

[B73] Sajjad W, Din G, Rafiq M, Iqbal A, Khan S, Zada S, Ali B, Kang S (2020) Pigment production by cold-adapted bacteria and fungi: colorful tale of cryosphere with wide range applications. Extremophiles 24: 447–473. 10.1007/s00792-020-01180-2 PMC7266124 32488508

[B74] Sánchez‐Ramírez S, Tulloss RE, Amalfi M, Moncalvo JM (2015) Palaeotropical origins, boreotropical distribution and increased rates of diversification in a clade of edible ectomycorrhizal mushrooms (*Amanita* section *Caesareae*). Journal of Biogeography 42: 351–363. 10.1111/jbi.12402

[B75] Singer R (1956) A fungus collected in the Antarctic. Sydowia-Beihefte 1: 16–23.

[B76] Smith AH (1953) New species of *Galerina* from North America. Mycologia 45: 892–925. 10.1080/00275514.1953.12024323

[B77] Smith AH, Singer R (1964) A monograph on the genus *Galerina* Earle. Hafner Publishing Company, New York.

[B78] So JE, Halda JP, Hong SG, Hur J-S, Kim JH (2023) The revision of lichen flora around maxwell bay, king george island, maritime antarctic. Journal of Microbiology 61: 159–173. 10.1007/s12275-023-00015-x PMC9998320 36847971

[B79] Stamatakis A (2014) RAxML version 8: a tool for phylogenetic analysis and post-analysis of large phylogenies. Bioinformatics 30: 1312–1313. 10.1093/bioinformatics/btu033 PMC3998144 24451623

[B80] Stuchlik L (1981) Tertiary pollen spectra from the Ezcurra Inlet Group of Admiralty Bay, King George Island (South Shetland Islands, Antarctica). Studia Geologica Polonica 72: 109–132.

[B81] Vences M, Miralles A, Brouillet S, Ducasse J, Fedosov A, Kharchev V, Kostadinov I, Kumari S, Patmanidis S, Scherz MD, Puillandre N, Renner SS (2021) iTaxoTools 0.1: Kickstarting a specimen-based software toolkit for taxonomists. Megataxa 6: 77–92. 10.11646/megataxa.6.2.1

[B82] Vila J (2006) Noves dades sobre el component fúngic de les comunitats de Cistus de Catalunya, II. Revista Catalana de Micologia 28: 167–207.

[B83] Vila J, Llimona X (2009) Noves dades sobre el component fúngic de les comunitats de Cistus de Catalunya. III. Addicions, correccions i claus d’identificació. Revista Catalana de Micologia 31: 103–137.

[B84] Vilgalys R, Hester M (1990) Rapid genetic identification and mapping of enzymatically amplified ribosomal DNA from several *Cryptococcus* species. Journal of bacteriology 172: 4238–4246. 10.1128/jb.172.8.4238-4246.1990 PMC213247 2376561

[B85] Voitk A, Saar I, Burzynski M, Corriol G (2024) The *Arrhenia peltigerina* complex—preliminary report. Botany 102: 248–267. 10.1139/cjb-2023-0138

[B86] Zhang J, Kapli P, Pavlidis P, Stamatakis A (2013) A general species delimitation method with applications to phylogenetic placements. Bioinformatics 29: 2869–2876. 10.1093/bioinformatics/btt499 PMC3810850 23990417

[B87] Zhao R-L, Li G-J, Sánchez-Ramírez S, Stata M, Yang Z-L, Wu G, Dai Y-C, He S-H, Cui B-K, Zhou J-L (2017) A six-gene phylogenetic overview of *Basidiomycota* and allied phyla with estimated divergence times of higher taxa and a phyloproteomics perspective. Fungal Diversity 84: 43–74. 10.1007/s13225-017-0381-5

[B88] Zhao R-L, Zhou J-L, Chen J, Margaritescu S, Sánchez-Ramírez S, Hyde KD, Callac P, Parra LA, Li G-J, Moncalvo J-M (2016) Towards standardizing taxonomic ranks using divergence times–a case study for reconstruction of the *Agaricus* taxonomic system. Fungal Diversity 78: 239–292. 10.1007/s13225-016-0357-x

